# Tailoring selectivity and efficiency: pyrazolyl-1*H*-1,2,4-triazole **MCM-41** and silica hybrid materials for efficient cadmium(II) removal from water

**DOI:** 10.1007/s11356-025-36353-z

**Published:** 2025-04-05

**Authors:** Youssef Draoui, Smaail Radi, Amal El Mahdaoui, Mohamed El Massaoudi, Aurelian Rotaru, Yann Garcia, Maria do Amparo F. Faustino, Maria da Graça P. M. S. Neves, Nuno M. M. Moura

**Affiliations:** 1 LCAE, Department of Chemistry, Faculty of Science, University Mohamed I, P.O. Box 524, 60 000 Oujda, Morocco; 2 Department of Electrical Engineering and Computer Science & Research Center Mansid, “Stefan Cel Mare” University, University Street, No. 13, 720229 Suceava, Romania; 3 Institute of Condensed Matter and Nanosciences, Molecular Chemistry, Materials and Catalysis (IMCN/MOST), Université Catholique de Louvain, Place L. Pasteur 1, 1348 Louvain-La-Neuve, Belgium; 4 LAQV-REQUIMTE, Department of Chemistry, University of Aveiro, 3810-193 Aveiro, Portugal; 5 Higher School of Education and Training, University Mohamed I, P.O. Box 410, 60000 Oujda, Morocco

**Keywords:** Hybrid materials, Immobilization, Water pollution, Remediation, Hazardous metal ions, Adsorption

## Abstract

Two novel silica-based hybrid materials, **M1** and **M2**, based on silica gel and **MCM-41** with a ditopic triazole-pyrazole ligand grafted onto their surfaces, respectively, were successfully synthesized and fully characterized. The adsorption capacity of these organic–inorganic hybrid materials was evaluated for copper(II), cadmium(II), and lead(II) ions. Experimental parameters including solution pH, contact time, temperature, and adsorbate concentration were systematically investigated. The obtained experimental data were analyzed using diverse adsorption isotherms and models to assess and interpret the behavior of the adsorbents. Notably, the newly synthesized materials exhibited exceptional selectivity, ultra-rapid adsorption rates within the first few minutes, and high removal efficiencies of 81.40 and 121.26 mg/g for **M1** and **M2**, respectively, towards Cd(II). Moreover, reusability assessments demonstrated excellent consistency, with only marginal decreases in adsorption capacities of less than 8% observed over five consecutive cycles. Interestingly, the application of **M1** and **M2** for the extraction of transition metals from real contaminated river water, from Nador city in Morocco, showcased their effectiveness in removing heavy metal ions even at low concentrations, with **M2** achieving up to 67% cadmium removal. These findings highlight the potential of both materials as reliable systems for heavy metal removal in practical environmental remediation applications, with material **M2** demonstrating superior performance over **M1**.

**Graphical abstract:**

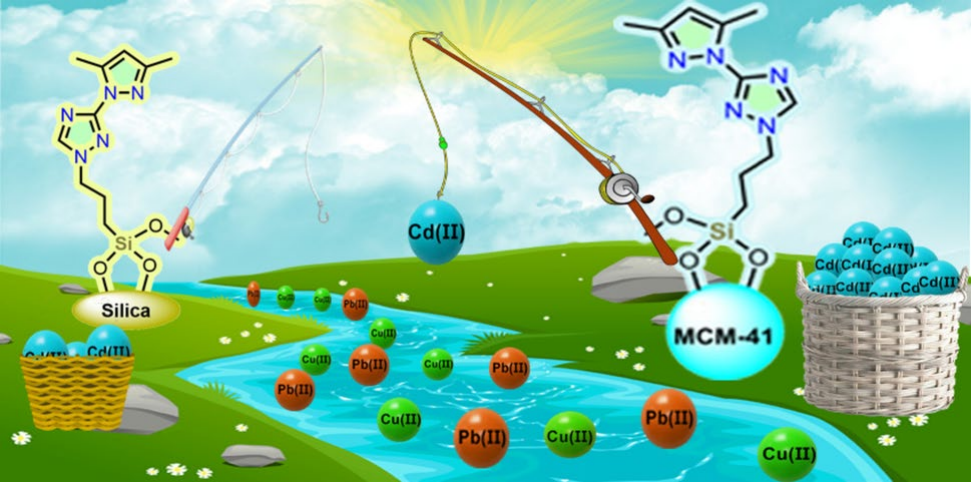

**Supplementary Information:**

The online version contains supplementary material available at 10.1007/s11356-025-36353-z.

## Introduction

Water is essential for the survival of all living organisms on Earth, and its accessibility significantly influences the global economy. Its availability is essential not only for human consumption but also for diverse sectors, including agriculture, industry, and recreational activities. Regrettably, one-sixth of the world’s population grapples with water pollution issues (Elimelech 2006), which can be categorized into two major forms of contamination: organic (Thomaidi et al. 2015) and inorganic (Srivastav and Ranjan 2020) pollutants. Inorganic pollution, even at low concentrations, poses considerable dangers, particularly when it involves heavy metals like cadmium and lead (Järup et al. 1998; Karri et al. 2016; Abedi Sarvestani and Aghasi 2019). Various technologies, such as membrane filtration, reverse osmosis, electro-dialysis, floatation, precipitation, electrochemical methods, flocculation-coagulation, and aerobic or anaerobic treatment, have been developed and implemented for water treatment (Lakherwal 2014; Ebbers et al. 2015; Litu et al. 2019; Wu et al. 2019). These techniques aim to address heavy metal removal concerns; however, challenges related to consistency, cost-effectiveness, selectivity, and efficient purification persist, prompting ongoing research efforts in this field (Zhu et al. 2019; Zamora-Ledezma et al. 2021; Qasem et al. 2021).

Extensive research efforts spanning several decades have focused on the development of efficient materials for environmental applications. Biomaterials, nanocomposites, and hybrid materials have garnered significant attention (Ramalingam et al. 2018; Milošević et al. 2021). Hybrid materials, in particular, offer numerous advantages due to their unique chemical and physicochemical properties, positioning them at the forefront of technological advancements. The combination of dissimilar components within a single material allows for a virtually boundless array of possibilities, resulting in the creation of new materials with multifunctional properties. These hybrid materials inherit advantageous characteristics from their inorganic components such as thermal stability and rigidity (Bershtein et al. 2002), while also incorporating the desirable traits of organic materials like flexibility, ductility, chelation, and ease of processing (Li et al. 2013; Radi et al. 2016), As a result, innovative applications across diverse fields have been made possible, paving the way for novel advancements.

One of the most appealing applications of these materials is their use as adsorbents in order to eradicate heavy metals in aqueous solutions (Huang et al. 2018; Faustini et al. 2018; Awual 2019). Silica-based materials, in particular, have recently attracted considerable interest and development (Radi et al. 2018; Tighadouini et al. 2019a; Rajendran et al. 2022). The silica framework offers exceptional thermal resilience, structural integrity, and a high surface area for adsorption, while the organic moieties contribute to enhanced selectivity, chelation properties, and the ability to tailor chemical interactions. This synergy allows silica-based hybrids to surpass traditional adsorbents. The properties and characteristics of these hybrid systems can be easily adjusted and enhanced based on the specific organic components incorporated. Remarkable improvements in adsorption capacity (Radi et al. 2019; El Abiad et al. 2023), rapid removal (El-Massaoudi et al. 2021), exceptional selectivity (Tighadouini et al. 2019b, 2022), and consistent reusability (Tighadouini et al. 2020) have been reported in numerous studies. Therefore, our primary objective is to synthesize silica-based adsorbents that encompass all these advantages, making them a reliable and environmentally friendly option for industrial-scale applications (Yu et al. 2016; Suhail et al. 2020).

In this context, two novel hybrid materials, **M1** and **M2**, were synthesized using 60 Å silica gel and **MCM-41** (mobile crystalline material), as the inorganic components, respectively, and 3-(3,5-dimethyl-1*H*-pyrazol-1-yl)−1*H*−1,2,4-triazole (**L**) previously synthesized (Draoui et al. 2023a, b) as the organic component. The design of these materials considers the high affinity of triazole-based heterocycles for transition metal ions, as well as the different structural features of **MCM-41** silica compared to 60 Å silica, including its highly ordered mesoporous structure, uniform pore size, and high surface area (Costa et al. 2020).

The new hybrid materials were thoroughly characterized applying various physical and spectroscopic techniques. The influence of several parameters, including the adsorbate concentration, contact time, temperature, and solution pH on their respective adsorption capacities, was individually investigated. Furthermore, the efficiency of new hybrid materials to be reused and their selectivity towards metal ions were also evaluated. To gain insight into the adsorption mechanism and facilitate effective result analysis, the Langmuir, Freundlich, Temkin, and Sips adsorption isotherm models, as well as the pseudo-first-order, pseudo-second-order, Elovich, and intraparticle diffusion kinetic models, were applied to the experimental data and discussed.

This study presents a detailed comparative analysis of silica and **MCM-41**, providing unique insights on the impact of surface structure and porosity in adsorption performance. The results revealed that both materials exhibited exceptional selectivity towards cadmium ions, a significant achievement given the typical co-adsorption of copper ions. This selectivity is particularly advantageous for industrial applications, since cadmium is a highly toxic metal and presents risks due to widespread environmental contamination. Achieving effective cadmium removal with minimal copper uptake is an uncommon accomplishment, as highlighted in recent research studies (Wang et al. 2016; Khalifa et al. 2020; Wan et al. 2021; Thirupathi et al. 2023). This distinction highlights the potential of these materials to offer precise, efficient, and cost-effective solutions for water treatment. The combination of exceptional cadmium selectivity, high adsorption capacity, rapid removal rates, and excellent reusability underscores and supports their suitability for addressing cadmium contamination in aqueous environments, meeting critical industrial and environmental needs.

## Experimental section

### Materials

All tests utilized double-distilled water (conductivity ≤ 1.5 µS/cm). Stock solutions of metal ions were prepared from commercial metal salts: Cd(NO₃)₂·6H₂O, Cu(NO₃)₂·3H₂O, and Pb(NO₃)₂·6H₂O. Solvents were all of analytical grade and with purity greater than 99.5% and were used without further purification. When necessary, they were dried according to established protocols. The chemicals 3-amino-1,2,4-triazole (C_2_H_4_N_4_, ≥ 95%), L-( +)-ascorbic acid (C_6_H_8_O_6_, ≥ 99%), sodium nitrite (NaNO_2_, ≥ 98%), sodium iodide (NaI, ≥ 99%), (3-chloropropyl)trimethoxysilane (C_6_H_15_O_3_SiCl, ≥ 97%), and triethylamine (C_6_H_15_N, ≥ 99.5%) were purchased as analytical grade and used without further purification. Commercial technical-grade silica gel (60 Å, 70–230 mesh) and **MCM-41** with a 0.98 cm^3^/g pore volume (hexagonal) were used for the synthesis of hybrid materials **M1** and **M2**, respectively. Real contaminated water samples were collected from the Marchica river, located at GPS coordinates 535Q + WGR in Nador, Morocco.

### Silica-based material preparation

#### Synthesis of Si-Cl

The functionalized silica **Si-Cl** with CPTMS (3-chloropropyltrimethoxysilane) was carried out by conducting the following procedure in accordance with the literature (Prado and Airoldi 2001; Sales et al. 2004): 2 g of silica gel technical grade with a pore size of 60 Å, 70–230 mesh was first activated in an oven at a temperature of 120 °C for 12 h. Then, to the activated silica in 50 mL of anhydrous toluene, 1 mL of CPTMS was added. The resulting mixture was refluxed under a nitrogen atmosphere at 110 °C for 24 h. Afterward, the reaction mixture was filtered, and the solid obtained was washed three times with 5 mL each of toluene, methanol, and dichloromethane. The solid was subsequently dried in an oven at 50 °C for 6 h to yield the functionalized silica **Si-Cl**.

#### Synthesis of MCM-41-Cl

The functionalized mesoporous **MCM-41-Cl** was also prepared following a documented procedure (De Lima et al. 2014). Two grams of mesostructured silica **MCM-41** with 0.98 cm^3^/g pore volume was activated in an oven at a temperature of 120 °C for 12 h. Then, 1 mL of CPTMS was added to the activated **MCM-41** in 80 mL of dried toluene under a nitrogen atmosphere. After 2 h under reflux, approximately 5 mL of toluene was distilled off, and then, 2 mL of CPTMS was added to the mixture. This cycle was repeated three times, with the third iteration involving the addition of 4 mL of 3-chloropropyltrimethoxysilane. After 48 h, the resulting mixture was filtered, and the solid obtained was washed three times, starting with 5 mL toluene, followed by methanol and then dichloromethane. Finally, the material was dried in an oven at 50 °C for 6 h to yield the functionalized **MCM-41-Cl**.

#### Synthesis of the 3-(3,5-dimethyl-1H-pyrazol-1-yl)−1H-1,2,4-triazole, L

The synthesis of the organic counterpart **L** was previously reported by our group as follows (Draoui et al. 2023b). A water solution containing 5.3 g of sodium nitrite (76.82 mmol) was added dropwise to a continuously stirred solution composed of 5.38 g of 1*H*−1,2,4-triazol-3-amine (64.01 mmol) dissolved in 36 mL of 12 M hydrochloric acid, at a temperature of 0 °C. After stirring for 1 h, 11.28 g of ascorbic acid (64.04 mmol) dissolved in 54 mL of cold water was added slowly, while maintaining the 0 °C temperature. Following 10 min of stirring, a solution consisting of 6.4 g of acetylacetone (64.04 mmol), along with a few drops of ethanol, was slowly introduced into the reaction. The mixture was then left to stir at room temperature for 1 day.

The resulting mixture was subsequently neutralized to a pH of 7.0 using sodium carbonate. The aqueous phase was extracted three times with 20 mL portions of chloroform, then dried using magnesium sulfate, and finally evaporated to remove the solvent. To the resulting mixture, 30 mL of diethyl ether was added, and it was left at a temperature of 0 °C overnight. After filtration, **L** was obtained as a white powder in 40% yield. The analytical data agrees with that reported in the literature (Draoui et al. 2023b).

#### Synthesis of M1 and M2

To 2 g of each functionalized silica, **Si-Cl** and **MCM-41-Cl**, in 50 mL of dry toluene, 0.5 g and 1 g of **L** were added, along with 0.4 mL and 0.8 mL of triethylamine, and 0.5 and 1 g of NaI, respectively. The reaction was carried out for 48 h at 110 °C under nitrogen atmosphere. The resulting mixture was filtered, and the solids obtained were washed three times with 5 mL of toluene, methanol, and dichloromethane, then dried in an oven for 6 h at 50 °C to give the hybrid materials **M1** and **M2**, respectively.

### Studies of the adsorption efficiency of M1 and M2 for metal Ions

The adsorption of metal ions onto the newly prepared adsorbents **M1** and **M2** was conducted through a batch experiment. In each case, the adsorption tests were repeated three times, and only the averaged outcome was reported. After adsorption, the solid phase was isolated by filtration using a 0.45-mm syringe filter. The concentration of the remaining metal ions in the filtrate was subsequently determined using an atomic absorption spectrophotometer.

The adsorption capacity of each hybrid towards Cd(II), Cu(II), and Pb(II) was calculated using the following equation:

$${q}_{\text{e}}=\left({C}_{0}-{C}_{\text{e}}\right)\frac{V}{m}$$

Here, *q*_e_ represents the amount of each metal ion on the adsorbent (mg/g), *C*_0_ is its initial concentration (mg/L), *C*_e_ is the equilibrium concentration in solution (mg/L), *V* is the volume of metallic solution (L), and *m* is the weight of the adsorbent (g).

The impact of each parameter on the adsorption capacity of each material **M1** and **M2** was evaluated individually as follows:

- Initial concentration effect: 10 mL of a solution containing 10, 30, 60, 90, 120, 150, 180, 210, 260, and 300 mg/L of each metal ion (Cd(II), Cu(II), and Pb(II)) was introduced into a conical flask holding 10 mg of each adsorbent **M1** or **M2**. The mixture was stirred for 2 h, while keeping the temperature at 298 K and pH fixed at 6.0. The concentration of each metal ion in the filtrate was examined according to the procedure referred above. The optimal concentration determined from this experiment was 180 mg/L.
- Contact time effect: A conical flask containing 10 mg of each hybrid material was used. Then, 10 mL of an aqueous solution containing the metal ion at the optimum concentration (180 mg/L) was added. The concentration of each metal ion in the filtrate was examined after the following contact times: 5, 10, 15, 20, 25, 30, 45, and 60 min. All assays were performed at 298 K and pH 6.0. The optimal contact time determined from this experiment was 30 min.
- Thermodynamic survey: To a conical flask with 10 mg of each adsorbent, 10 mL of an aqueous solution containing the metal ion at the optimal concentration (180 mg/L) was added. The concentration of each metal ion in the filtrate was examined at temperatures of 298 K, 308 K, and 318 K, while maintaining the other parameters at their optimal values (contact time 30 min and pH 6.0).
- pH variation impact: 10 mg of each adsorbent was added to a conical flask containing an aqueous solution with the metal ion at an optimal concentration, with the pH adjusted at values of 3, 4, 5, 6, and 7. The analysis of the metal ions in the filtrate was performed after maintaining the material in contact with the analyte for 30 min at 298 K. The pH adjustments were performed using a pH meter and diluted solutions of HCl and NaOH. The optimal pH determined from this experiment was 6.0.
- Selectivity: 10 mg of each adsorbent was added to a conical flask containing a mixture of the three metal ions: Cd(II), Cu(II), and Pb(II) (180 mg/L each) in 10 mL of an aqueous solution. The analysis of the metal ions in the filtrate was performed after maintaining each material in contact with the analytes for 30 min at 298 K and pH 6.0.
- Reusability: In a round-bottom flask containing 10 mL of an aqueous solution of nitric acid (4%), 1 g of each adsorbent previously loaded with the metal ion was added. The mixture was stirred for 2 h, filtered, and then washed with water until the pH of the filtrate was neutral. The solid was dried, and then, 10 mg was reused for each metal ion. This process was repeated five times, and the adsorption capacity of the adsorbent after each cycle was analyzed.
- Real water decontamination: 10 mL of Marchica water samples was introduced to a conical flask containing 10 mg of the adsorbent. The analysis of the metal ions was performed after maintaining each material in contact with the mixture for 30 min at 298 K.

Additionally, the same samples were loaded with 180 mg/L of each metal ion (Cd(II), Cu(II), or Pb(II)). The adsorption analyses were performed after keeping each absorbent in the presence of the mixture for 30 min at 298 K and with the pH fixed to 6.0.

## Result and discussion

### Synthesis of the adsorbents

**Scheme 1:**
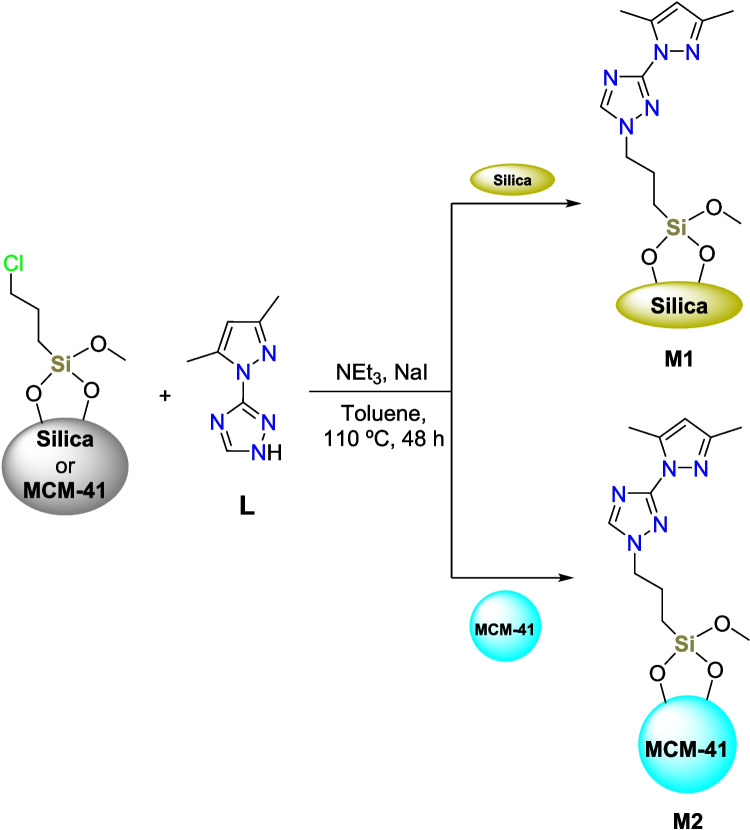
Synthesis of **M1** and **M2** from the 60 Å silica gel (**Si**) and **MCM-41** functionalized with 3-chloropropyltrimethoxysilane (CPTMS)

The newly synthesized hybrid materials **M1** and **M2** were prepared according to the procedure summarized in Scheme 1. The synthesis was carried out in two straightforward steps: the functionalization of 60 Å silica (**Si**) and **MCM-41** with 3-chloropropyltrimethoxysilane (CPTMS), followed by the grafting of the selected ligand **L** in the presence of triethylamine and sodium iodide. This ligand was obtained through the diazotization of 3-amino-1,2,4-triazole, followed by reaction with acetylacetone in the presence of ascorbic acid. The ligand **L** was obtained as a white powder by precipitating from a diethyl ether solution with 40% yield (Draoui et al. 2023a, b).

The precise amounts of the functionalization agent and grafted ligand **L** were controlled using thin layer chromatography (TLC) until maximum immobilization was achieved. This method revealed that the **MCM-41** support required nearly double the amount of the organic components compared to standard 60 Å silica gel (**Si**). Therefore, we anticipate that the newly developed **MCM-41**-based adsorbent **M2** will outperform silica-based adsorbent **M1**. Moreover, and as mentioned above, our previous work demonstrated that the ligand **L** has strong coordination capabilities with a variety of transition metals making **L** a promising candidate for efficient heavy metal extraction (Draoui et al. 2023a, b).

### Hybrid material structural characterization

#### Attenuated total reflection-Fourier transform infrared (ATR-FTIR) spectra analysis

**Fig. 1:**
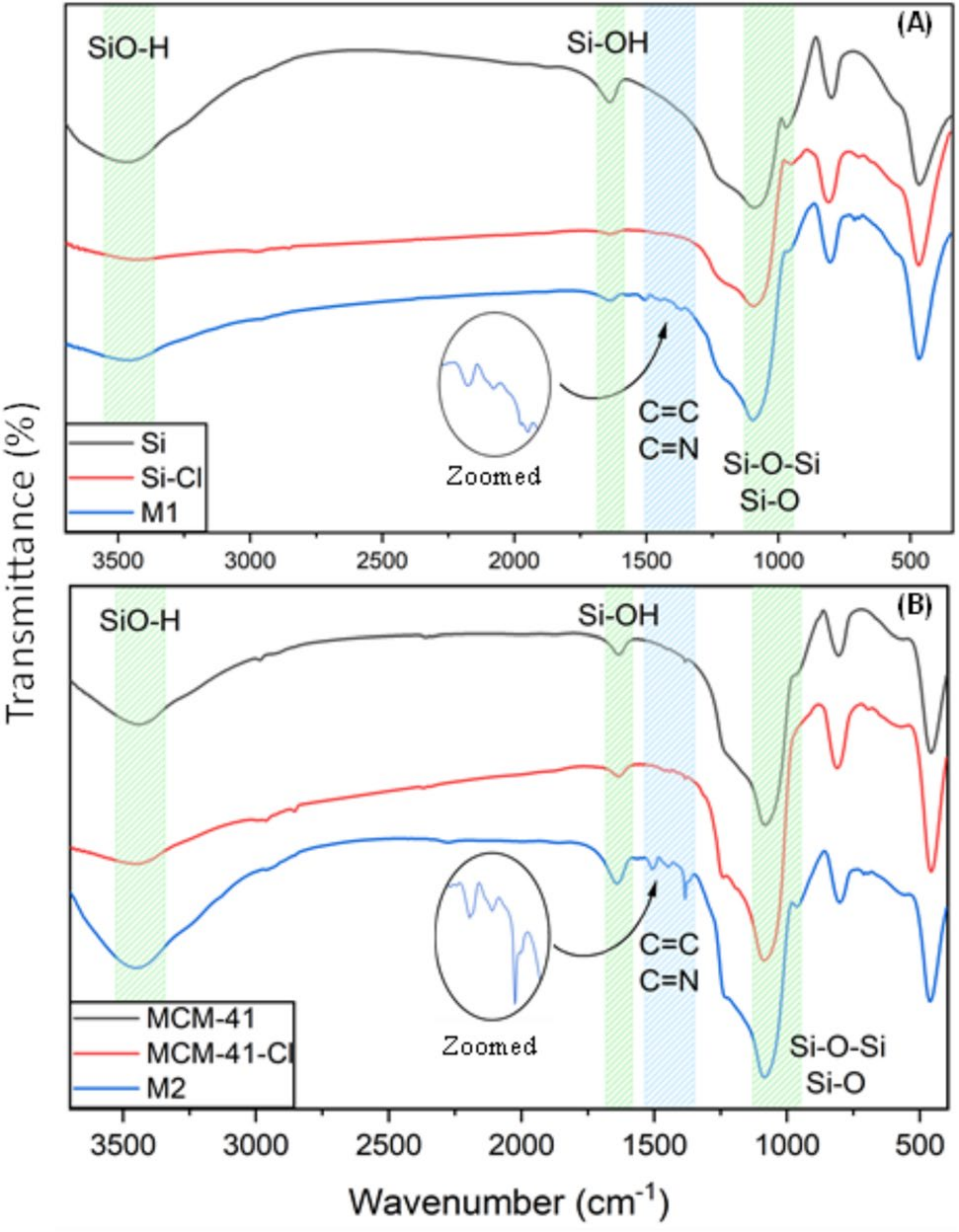
ATR-FTIR spectra of **M1** (**A**) and **M2** (**B**) and of the corresponding precursors. Insets show a zoom between 1600 and 1300 cm^−1^

The silica gel **Si** and **MCM-41** display characteristic broad and intense bands corresponding to SiO–H stretching vibrations around 3470 cm^−1^ and Si–OH vibrations near 1630 cm^−1^ (Suhail et al. 2020). Additionally, the prominent bands at approximately 1090 cm^−1^ and 970 cm^−1^ are attributed to Si–O–Si stretching and Si–O vibrations, respectively (Mutneja et al. 2016). The functionalization of **Si** and **MCM-41** with CPTMS has almost no impact on the general appearance of the ATR-FTIR spectra of the resulting materials **Si-Cl** and **MCM-41-Cl** (Fig. 1). However, following the immobilization of the triazole ligand **L**, noticeable new peaks emerged in the 1380 to 1570 cm^−1^ range in both spectra of the resulting materials **M1** and **M2**. These peaks indicate the appearance of C = C stretching vibrations and C = N aromatic groups, both of which are present in the triazole branches, thereby confirming the success of the covalent grafting of **L** onto the silica gel. Furthermore, it is notable that these spectral bands appear more pronounced in the case of **M2**. This could be ascribed to the larger specific surface area of **MCM-41**, which potentially facilitated a higher percentage of grafting.

#### X-ray diffraction analysis

Figure OR1 (Online Resource) presents the XRD patterns of the materials and their precursors. For both adsorbents, the diffraction peak at (110) exhibits a noticeable decrease in intensity compared to the precursors. This change suggests that the incorporation of organic groups impacts the pore structure. The diminished peak intensity is likely due to the reduced silica content following immobilization. The increased triazole content, which contributes to this silica reduction, further supports the observed decline in intensity. These findings align with the successful modification of silica gel by the organic ligands (El Abiad et al. 2023).

#### Solid-state ^13^C NMR spectroscopy

The solid-state ^13^C NMR analysis also reveals the emergence of new peaks following the functionalization and modification of the silica. Both the **Si-Cl** and **MCM-41-Cl**-based adsorbents exhibit similar ^13^C NMR spectra, as depicted in Figure OR2 (Online Resource). These functionalized silica-based materials show signals in the aliphatic region around *δ* 8, 22, and 46 ppm, attributed respectively to the carbon resonances of the Si-CH_2_-CH_2_-CH_2_Cl chains (Figure OR2, Online Resource). Upon introduction of ligand **L**, two additional signals appear at approximately *δ* 144.5 and 150.5 ppm, which are assigned to the aromatic carbon resonances of the triazole and pyrazole rings. Conversely, the ^13^C NMR signal located around *δ* 50.8 ppm is primarily associated with non-substituted methoxy groups (-OCH_3_).

#### Thermogravimetric analysis

The thermal stability of the newly developed hybrid materials **M1** and **M2** was assessed via thermogravimetric analysis (TGA) and compared with that of their respective functionalized and non-functionalized precursors (Figure OR3, Online Resource). All hybrid materials revealed weight loss between 25 and 100 °C, which is attributed to the reduction of adsorbed water particles within the silica matrix (Arakaki et al. 2013). Furthermore, beyond 110 °C until reaching a temperature of 800 °C, **Si** and **MCM-41** experienced a respective mass reduction of 3.52% and 3.87%. This outcome is mostly assigned to the degradation of the condensed free silanol groups linked to the surface (Pérez-Quintanilla et al. 2007; Zhang et al. 2009). On the other hand, the functionalized precursors **Si-Cl** and **MCM-41-Cl** experienced much higher weight loss of 9.30% and 9.61%, respectively, due to the decomposition of the organic groups introduced on the silica. Besides, **M1** and **M2** exhibited an additional mass loss of 17.55% and 14.43%, respectively, which further proves the immobilization of the triazole ligand **L** onto silica. Importantly, it is noteworthy that these newly synthesized adsorbents exhibit remarkable thermal stability, as most of the material decomposition occurred at temperatures above 310 °C.

#### Elemental analysis

Elemental analysis plays a pivotal role in determining whether the organic component has been successfully bonded to the support surface. Table 1 presents a summary of the changes in carbon, hydrogen, and nitrogen content during the synthesis of the hybrid materials **M1** and **M2**. Prior to the functionalization of silica (**Si** and **MCM-41**), the elemental analysis indicated nearly negligible levels of carbon and nitrogen, which was anticipated given that silica primarily consists of SiO_2_. Subsequently, following the reaction with the immobilization agent CPTMS, the carbon content exhibited a substantial increase, reaching 5.662% and 7.648% for **Si-Cl** and **MCM-41-Cl**, respectively, while nitrogen levels remained negligible. Finally, with the introduction of the ligand **L**, nitrogen content also rose due to the incorporation of organic branches rich in nitrogen.

**Table 1:** Results of the elemental analysis of adsorbents **M1** and **M2** and their respective precursors

Sample	C%	H%	N%
**Si**	0.054	0.726	0.085
**Si-Cl**	5.662	1.598	0.045
**M1**	7.642	1.480	3.225
**MCM-41**	0.054	0.648	0.100
**MCM-41-Cl**	7.648	1.756	0.064
**M2**	8.392	1.526	4.589

#### Surface morphology

**Fig. 2:**
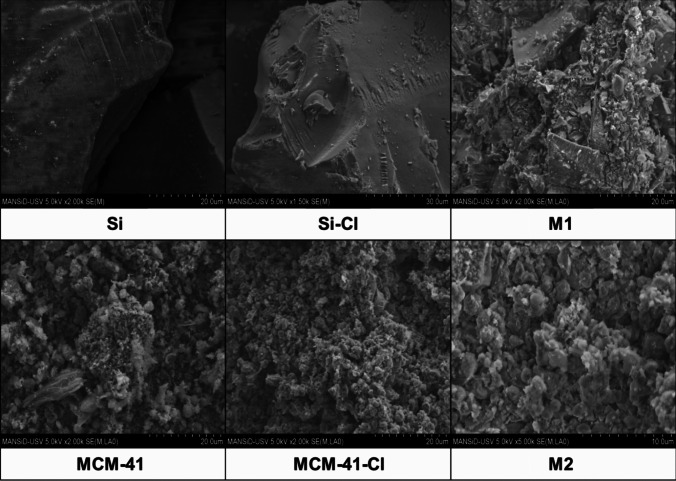
Scanning electron microscopy (SEM) of **M1** (top), **M2** (bottom), and their precursors

The alterations in morphological characteristics resulting from the functionalization and modification of the silica surface were examined using a scanning electron microscope (SEM). Figure 2 illustrates the surface images of **M1** and **M2** along with non-functionalized (**Si** and **MCM-41**) and functionalized precursors (**Si-Cl** and **MCM-41-Cl**). Initially smooth, the surface of native silica (**Si**) became relatively rough following functionalization with CPTMS. Additionally, the surface of **M1** with the attached **L** exhibited noticeable aggregation attributed to the high concentration of organic components. In contrast, the texture of **MCM-41** appears cottony and cloudy, distinct from that of the **Si** silica surface. Post-functionalization with CPTMS, the surface of **MCM-41-Cl** became more disordered, losing its previous cloudy appearance. Furthermore, **M2** resulting from the modification of **MCM-41-Cl** with **L** reveals agglomerated spherical particles, which are a characteristic feature of mesoporous materials (Yan et al. 2009).

#### Surface area and pore volume size analysis

**Fig. 3:**
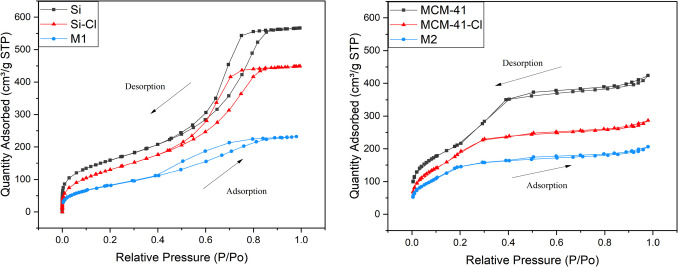
N_2_ adsorption–desorption isotherm of **M1** (left), **M2** (right), and their respective precursors

The values of the surface area (*S*_BET_) and pore volume (*V*_pore_) obtained from the Brunauer–Emmett–Teller (BET) surface area and Barrett-Joyner-Halenda (BJH) pore size analyses for **M1**, **M2**, and their precursors are compiled in Table 2. Figure 3 shows the N_2_ adsorption–desorption isotherms. According to the IUPAC classification, the curves exhibit a type IV profile, which is characteristic of mesoporous solids (Rouquerol et al. 1994). The isotherms, demonstrating uniform pore channels, were highly similar, suggesting complementary textural and framework-confined mesoporous systems.

**Table 2:** Physical surface parameters of materials **M1** and **M2** and their respective precursors

Materials	*S*_BET_ (m^2^/g)	*V*_pore_ (cm^3^/g)
**Si**	580.3158	0.56881
**Si-Cl**	490.5018	0.44092
**M1**	299.8736	0.43969
**MCM-41**	781.8610	0.79581
**MCM-41-Cl**	707.1388	0.57581
**M2**	343.1711	0.29890

As indicated in Table 2, the functionalization of **Si** with CPTMS led to a decrease in surface area from 580.3158 to 490.5018 m^2^/g, accompanied by a reduction in pore volume of *ca.* 22%. Moreover, the final modification of the functionalized silica **Si-Cl** with the organic ligand **L** resulted in a significant decrease in surface area and almost no alteration in pore size, with **M1** reaching 299.8736 m^2^/g. These declines are attributed to the introduction of triazole ligands onto the silica surface, which partially obstructs nitrogen molecule adsorption.

In the case of **MCM-41** series, the most significant reduction in surface area was observed in the final modification of **MCM-41-Cl** with the organic ligand **L** resulting in a decrease in *S*_BET_ from 707.1388 to 343.1711 m^2^/g for **M2**. A much less pronounced decrease, not exceeding 10%, was observed during the initial functionalization of **MCM-41**. In this series, both functionalizations were accompanied by a reduction in pore volume, from 0.79581 to 0.57581 cm^3^/g and then to 0.29890 cm^3^/g. This underscores the substantial potential for modification and ample space for alterations in **MCM-41** silica. In summary, the collective findings consistently indicate that as the surface undergoes modification, both the surface area and pore volume decrease.

### Studies of the adsorption efficiency of M1 and M2 for metal ions

#### Effect of concentration on adsorption and analysis of isotherms

**Fig. 4:**
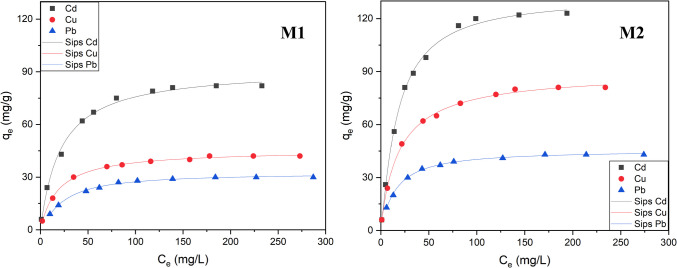
Effect of the initial concentration of each metal ion on the adsorption capacity of **M1** (left) and **M2** (right). Adsorption conditions: 10 mg of **M1** or **M2** in 10 mL of an aqueous solution containing each metal ion at a concentration ranging from 10 to 300 ppm, at 298 K, pH 6.0, and a contact time of 2 h. Experimental adsorption data fitted using Sips isotherm model

The capacity of a specific adsorbent to retain heavy metals is a pivotal characteristic of high-quality materials. In line with this, the relationship between the initial concentration of the selected metal ions, Cd(II), Cu(II), and Pb(II), and the adsorption capacity of **M1** and **M2** was examined. These assays were performed by stirring each hybrid in an aqueous solution (10 mg/10 mL) for 2 h at 25 °C and pH 6.0, with varying concentrations of each metal ion ranging from 10 to 300 ppm. Figure 4 shows the quantity of metal adsorbed (*q*_e_ mg/g) for each hybrid as a function of each equilibrium concentration (*C*_e_ mg/L).

**M1** exhibited a favorable extraction performance for all tested heavy metals (Cd(II), Cu(II), and Pb(II)) with particular success observed for Cd(II), reaching an impressive 81 mg/g of retained metal ion at an initial applied metal concentration of 180 mg/L (Fig. 4, left); the amount of removal for the other metal ions was 44 mg/L for Cu(II) and 30 mg/L for Pb(II). These results can be attributed to the presence of abundant nitrogen donors within the organic triazole-pyrazole branch **L** incorporated into the silica structure, which can effectively coordinate with various transition metals ions depending on the type and affinity of the ligand introduced.

Furthermore, the values significantly improved in the case of **M2**, achieving a remarkable 121 mg/g of removed Cd(II) at an initial applied metal concentration of 180 mg/L. Its efficiency to remove the other metal ions also improved attaining 83 mg/L for Cu(II) and 45 mg/L for Pb(II). The main difference between **M2** and **M1** is in the type of silica used since the extra chelating unit **L** is the same. These reported values affirm that the mesostructured silica-based hybrid material has a higher grafting percentage of ligands and likely shorter distances between each motif. This outcome was anticipated, as **MCM-41** offers a larger specific surface area, thus providing more opportunities for modification and the capture of metal ions.

To gain a deeper understanding of the adsorption types and mechanisms involved during the removal process, the four nonlinear adsorption isotherm equations, Langmuir, Freundlich, Temkin, and Sips, were fitted in the experimental data obtained. This allowed to identify the most appropriate and representative models for each equilibrium curve. Briefly, the Langmuir isotherm (Langmuir 1916) has been widely been used in the literature. The model involves both adsorption and desorption processes and assumes that the surface of solid adsorbent is homogeneous and associated with a monomolecular layer of adsorption. Moreover, the model claims that there is no interaction between adsorbed molecules. The equation of Langmuir isotherm is expressed as follows:

$${q}_{\text{e}}=\frac{{K}_{\text{L}}{Q}_{\text{L}}{C}_{\text{e}}}{1+{K}_{\text{L}}{C}_{\text{e}}}$$

where *C*_e_ is the equilibrium concentration (mg/L), *q*_e_ is the amount of metal adsorbed (mg/g), *Q*_L_ is the theoretical monolayer capacity (mg/g), and *K*_L_ is the sorption equilibrium constant (L/mg) related to the energy of adsorption.

Freundlich isotherm (Freundlich 1907) is one of the earliest documented relationships describing sorption equations. This model, unlike the Langmuir model, considers the solid adsorbent surface heterogeneous, allowing the occurrence of multi-molecular layer adsorption, and consequently interactions between adsorbed molecules may occur. Freundlich’s equation is purely empirical, giving comparatively a less satisfactory explanation of adsorption, since it does not establish a limit value or a clear saturation point. The Freundlich isotherm equation is stated as follows:

$${q}_{\text{e}}={K}_{\text{F}}{{C}_{\text{e}}}^{1/{n}_{F}}$$

where *K*_F_ represents the adsorption capacity (L/g) and *n*_F_ is the adsorption intensity.

The Temkin isotherm (Temkin 1940) analysis implies that all molecules’ adsorption heat decreases linearly with increasing adsorbent surface coverage, and that the adsorption has a maximum energy distribution of uniform bonds. The Temkin isotherm can be described by the following equation:

$${q}_{\text{e}}={B}_{\text{T}}\;\text{ln}\;{A}_{\text{T}}{C}_{\text{e}}$$

where *B*_T_ (*B*_T_ = *RT/b*_T_*)* (*b*_T_, Temkin isotherm constant) is related to the adsorption heat; meanwhile, *A*_T_ (L/g) is the equilibrium binding constant, corresponding to the maximum binding energy.

The Sips model (Sips 1948) is a hybrid form of the Langmuir and Freundlich models developed for forecasting adsorption in heterogeneous systems and ignoring the limitation of the Freundlich isotherm’s rising adsorbate concentration. So, at low adsorbate concentrations, the results match Freundlich isotherm but predict monolayer adsorption in a similar way to the Langmuir isotherm at high concentrations. As a result, the Sips isotherm is appropriate only to describe monolayer adsorption systems. The next equation expresses the Sips model:

$${q}_{\text{e}}=\frac{{K}_{\text{S}}{Q}_{\text{S}}{{C}_{\text{e}}}^{1/{n}_{S}}}{1+{K}_{\text{S}}{{C}_{\text{e}}}^{1/{n}_{S}}}$$

where *Q*_s_ is the theoretical Sips maximum adsorption capacity (mg/g), and *K*_s_ is the Sips equilibrium constant (L/mg). On the other hand, *1/ns* is the Sips model exponent.

Table 3 provides the values of the parameters obtained after fitting each of the previously mentioned isotherms to experimental data. From the analysis of the correlation coefficient *R*^2^ for each model, it becomes evident that both the Langmuir and Sips models exhibit the best fit and, consequently, serve as superior representative models. All obtained *R*^2^ values exceeded 0.990, reinforcing the notion that the mechanism can be elucidated by a monomolecular layer of adsorption. Furthermore, the adsorption process appears to be predominantly uniform, and as observed, the adsorption values eventually reach a saturation point with increasing concentration. These findings align with those reported for various silica−based materials (Najafi et al. 2011, 2012; Deng et al. 2020).

**Table 3:** Parameters of Langmuir, Freundlich, Temkin, and Sips isotherm adsorption models

Isotherm model	Adsorbent	**M1**			**M2**		
	Metal	Cd(II)	Cu(II)	Pb(II)	Cd(II)	Cu(II)	Pb(II)
Langmuir	*Q*_L_ (mg/g)	91.90	45.38	33.92	139.29	88.54	46.33
	*K*_L_	0.047	0.053	0.039	0.051	0.054	0.064
	*R*^2^	0.995	0.998	0.991	0.995	0.998	0.996
Freundlich	*n*_F_	3.257	3.636	3.699	3.066	3.362	4.309
	*K*_F_	17.30	9.95	7.26	25.03	17.88	12.90
	*R*^2^	0.921	0.911	0.870	0.904	0.927	0.871
Temkin	*B*_T_	15.65	8.02	6.62	25.15	15.18	8.13
	*A*_T_	1.075	0.973	0.506	0.921	1.176	1.156
	*R*^2^	0.967	0.981	0.945	0.967	0.981	0.944
Sips	*n*_S_	0.846	1.008	0.998	0.836	1.052	0.928
	*Q*_S_ (mg/g)	91.84	45.48	32.25	132.68	90.07	45.46
	*K*_S_	0.047	0.045	0.024	0.036	0.060	0.054
	*R*^2^	0.994	0.998	0.995	0.996	0.998	0.997

#### Effect of the time on adsorption and kinetic models

**Fig. 5:**
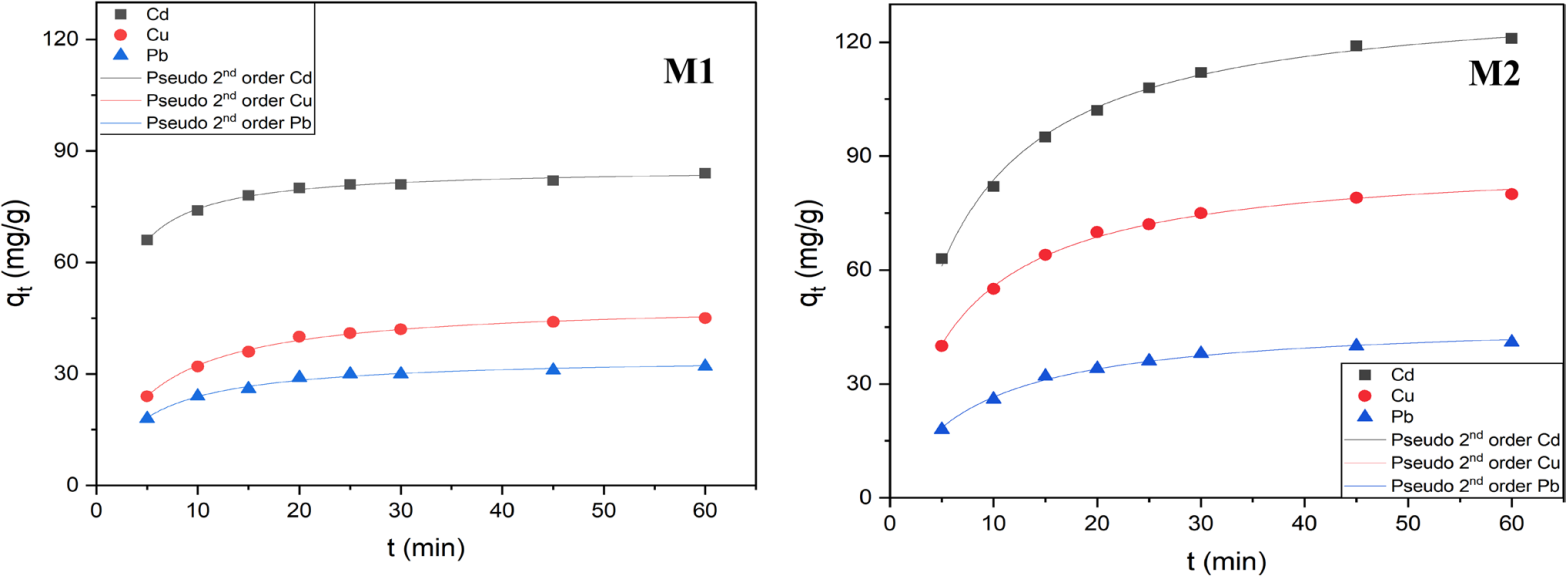
Effect of contact time on the adsorption rate of **M1** (left) and **M2** (right). Adsorption conditions: 10 mg of **M1** or **M2** in 10 mL of an aqueous solution containing each metal ion (180 mg/L) at pH 6.0 and 298 K for periods varying between 5 and 60 min. Experimental adsorption data fitted using the pseudo-second-order model

Assessing the kinetics of the adsorption process is crucial for evaluating the quality and reliability of an adsorbent. This analysis helps determine how quickly a substance can remove transition metals, thereby indicating its effectiveness and trustworthiness. Therefore, the effect of contact time on the adsorption rate of **M1** and **M2** was analyzed, and the results obtained are summarized in Fig. 5. These analyses were performed at 298 K and pH 6.0 with each material in contact with the optimal concentration of each metal ion (180 mg/L) for periods ranging from 5 to 60 min.

The results revealed that the silica-based material **M1** exhibits an extremely rapid adsorption for all adsorbates, with adsorption rates surging during the initial 5 min. Subsequently, adsorption marginally increases over time before reaching its peak value at 25 min. The **MCM-41**-based hybrid material **M2**, with an overall higher adsorption capacity, requires more time to reach the maximum adsorption (45 min). This adsorption delay might be primarily attributed to **M2**’s smaller pore sizes, necessitating an extended contact time between all adsorbates and the materials. This experiment highlights a trade-off between the advantage of higher capacity and a longer adsorption time.

To elucidate the mechanisms associated with the kinetics of the adsorption process for each material, a theoretical analysis of the data obtained was undertaken using representative models for adsorption processes: the pseudo-first-order, pseudo-second-order, and intraparticle diffusion models.

In the pseudo-first order (Lagergren 1898), it is assumed that the reaction rate is determined by the concentration of the metal ions since the second component, water, is in high excess and remains unchanged during the adsorption process. The kinetic model is conceived to be defined by physisorption and is expressed by the following equation:

$${q}_{\text{t}}={Q}_{1}(1-{e}^{-{K}_{1}t})$$

where *q*_t_ and *Q*_1_ are the adsorption capacity at the time *t* and equilibrium, respectively (mg/g), and *K*_1_ (min^−1^) is the rate constant of pseudo-first-order adsorption.

In the pseudo-second-order model (Ho and McKay 1999), it is assumed that the sorption rate decreases linearly as the adsorption process progresses, with a chemical reaction between the adsorbent and the adsorbate being considered the rate-determining step. This chemisorption can occur through two competitive, reversible second-order reactions at higher sorbate/sorbent ratios and a reversible second-order reaction at lower ratios. This model can be expressed by the following equation:

$${q}_{\text{t}}=\frac{{K}_{2}{{Q}_{2}}^{2}t}{1+{K}_{2}{Q}_{2}t}$$

where *q*_t_ and *Q*_2_ are the adsorption capacities at the time *t* and equilibrium, respectively (mg/g), and *K*_2_ (min^−1^) is the rate constant of pseudo-second-order adsorption.

The Elovich model (Chien and Clayton 1980) is often employed to describe chemisorption processes in which the adsorption rate decreases over time due to progressive saturation of surface sites. This model is particularly effective for systems involving heterogeneous adsorbent surfaces. The model is expressed by the following equation:

$${q}_{t}=\frac{1}{\beta }\text{ln}(1+\alpha \beta t)$$

where *α* (g mg^−1^ min^−1^) is the Initial adsorption rate constant, representing the adsorption rate at *t* = 0, while *β* (g/mg) is the desorption constant, related to the degree of surface coverage and the activation energy of associated with the chemisorption process.

Finally, the intraparticle diffusion model (Weber and Morris 1963) that has been usually used to identify diffusion mechanism in the adsorption processes involves two steps: surface diffusion and pore diffusion. The porosity, distribution, and morphology of particles are critical aspects impacting the pore diffusion. We purposefully used this model to inspect how porosity affects the differences in adsorption rates between silica and mesostructured silica-based hybrid materials. The equation can be expressed as follows:

$${q}_{\text{t}}={K}_{\text{id}}{t}^{1/2}+C$$

where *K*_id_ is the diffusion rate constant (mg min^1/2^/g) and *C* is a constant (mg/g) that provides information about the thickness of the boundary layer. The higher the *C* value, the greater the effect of the boundary layer. The plot of *q*_t_ as a function of *t*^1/2^ should represent a straight line; however, nonlinearity may also be found due to the possibility of multiple processes controlling the overall rate.

Table 4 provides details on the parameters obtained after fitting the aforementioned kinetic models to the experimental data, and it includes the values of the correlation coefficient *R*^2^ for each kinetic model. Concerning the correlation coefficients *R*^2^, it is evident that the pseudo-second-order model proved to be the appropriate fit for these results, consistently yielding values higher than 0.990. Furthermore, it is noteworthy that the correlation coefficient for intraparticle diffusion was higher in **MCM-41**-based material **M2** compared to silica-based material **M1**. Nevertheless, despite this observation, the most suitable fitting model for **M2** remains the pseudo-second order, since both types of silicates undergo surface modifications.

**Table 4:** Kinetic parameters of pseudo-first-order, pseudo-second-order, Elovich models for adsorption processes, and intraparticle diffusion models

Kinetic models	Adsorbent	**M1**			**M2**		
	Metal	Cd(II)	Cu(II)	Pb(II)	Cd(II)	Cu(II)	Pb(II)
Pseudo 1st order	*Q*_1_ (mg/g)	89.54	41.54	29.99	119.43	80.38	42.07
	*K*_1_	0.435	0.203	0.238	0.107	0.115	0.121
	*R*^2^	0.829	0.947	0.890	0.984	0.981	0.974
Pseudo 2nd order	*Q*_2_ (mg/g)	85.48	45.61	32.50	133.38	89.47	46.01
	*K*_2_	0.007	0.007	0.013	0.001	0.002	0.002
	*R*^2^	0.993	0.992	0.996	0.996	0.996	0.994
Elovich	*α*	9.77	202.44	321.20	43.14	35.44	24.23
	*β*	0.25	0.17	0.26	0.03	0.05	0.11
	*R*^2^	0.908	0.903	0.895	0.950	0.946	0.966
Intraparticle diffusion	*C*	71.45	27.00	21.12	42.87	31.70	17.58
	*K*_id_	1.612	2.405	1.500	11.688	7.508	3.811
	*R*^2^	0.753	0.758	0.787	0.852	0.841	0.841

In summary, all the materials under examination adhere to a chemisorption process, signifying the presence of chemical interactions. Metal ions stick to the adsorbent surface by forming chemical bonds and tend to occupy sites that maximize their coordination with the surface.

#### Thermodynamic studies

**Fig. 6:**
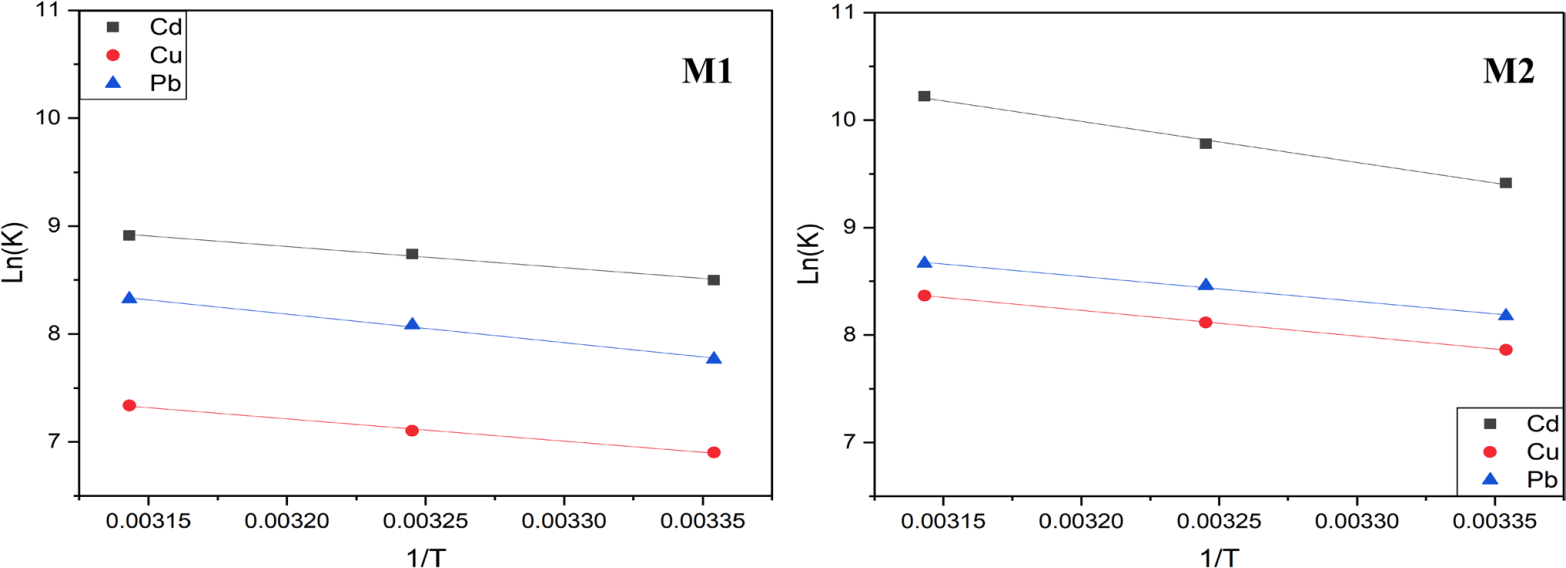
Effect of temperature on metal ions sorption by **M1** and **M2**. Adsorption conditions: 10 mg of **M1** or **M2** in 10 mL of an aqueous solution containing the metal ion at its optimal concentration (180 mg/L), at pH 6.0, for 30 min, and at temperatures 298, 308, and 318 K

The evaluation of thermodynamic parameters like enthalpy Δ*H*, entropy Δ*S*, and Gibbs free energy Δ*G* can also help understand the adsorbent behavior and the impact of temperature. A negative Δ*H* indicates an exothermic process, while a positive Δ*H* shows an endothermic process, with higher temperatures enhancing adsorption. Negative Δ*S* suggests a less random adsorption, whereas a positive Δ*S* points to more randomness. For Δ*G*, a negative value means adsorption is favorable and spontaneous, becoming more favorable at higher temperatures, while a positive Δ*G* indicates non-spontaneous adsorption. For each hybrid, the thermodynamic parameters were evaluated by performing the adsorption experiments at temperatures 298 K, 308 K, and 318 K in the presence of each metal ion at its optimum concentration (180 mg/L) and at pH 6.0 (Fig. 6). The values were calculated using the following equations according to the X. Zhou method (Zhou and Zhou 2014):

$$K={K}_{\text{d}} \times {M}_{\text{adsorbate}} \times \gamma$$

$$\text{ln}K=-\frac{\Delta H}{RT}+\frac{\Delta S}{R}$$

$$\Delta G=\Delta H-T\Delta S$$

where *K*_d_ (L/g) is the experimental distribution constant, *K* is the equilibrium constant, *γ* is the coefficient of activity, and *R* (8.314 J mol^−1^ K^−1^) is the ideal gas constant.

The values of Δ*H*, Δ*S*, and Δ*G* summarized in Table 5 were calculated by plotting ln*K* vs 1/*T* as it is represented in Fig. 6. The positive value of Δ*H* indicates endothermic sorption, which explains the rise in adsorption capabilities with higher temperatures for all metal ions. An increase in the randomness at the adsorbate/adsorbent interface is confirmed by the positive value of Δ*S*. Furthermore, the negative value of Δ*G* indicated that the adsorption reaction was spontaneous and feasible at ambient temperature. Additionally, the decrease of this parameter with rise in temperature stipulates that the adsorption of heavy metals was more favorable at higher temperature and the active sites on the surface of materials were more easily accessible.

**Table 5:** Thermodynamic parameters of **M1** and **M2**

Adsorbents	Metals	∆*H*° (kJ mol^−1^)	∆*S*° (kJ K^−1^ mol^−1^)	∆*G*° (kJ mol^−1^)		
				298 K	308 K	318 K
**M1**	**Cd(II)**	16.3721	0.1256	− 21.09	− 22.34	− 23.60
	**Cu(II)**	17.1872	0.1150	− 17.09	− 18.24	− 19.39
	**Pb(II)**	21.9746	0.1384	− 19.28	− 20.66	− 22.04
**M2**	**Cd(II)**	31.7922	0.1848	− 23.30	− 25.15	− 26.99
	**Cu(II)**	19.9321	0.1322	− 19.48	− 20.81	− 22.13
	**Pb(II)**	19.2828	0.1327	− 20.30	− 21.62	− 22.95

#### Effect of pH

Developing materials capable of effectively extracting heavy metals, under different pH conditions, remains a substantial challenge and a significant industrial objective. Thus, considering that the pH of a solution represents one of the primary factors that significantly influence the adsorption capacity of a given adsorbent, as indicated by previous studies (Wang et al. 2015; Da’na 2017), the presence of H^+^ ions not only has the potential to alter the surface charge of the adsorbent but also to impact the speciation of metal ions within the solution. Furthermore, H^+^ ions can engage in competition with metal ions when forming complexes with functional groups.

**Fig. 7:**
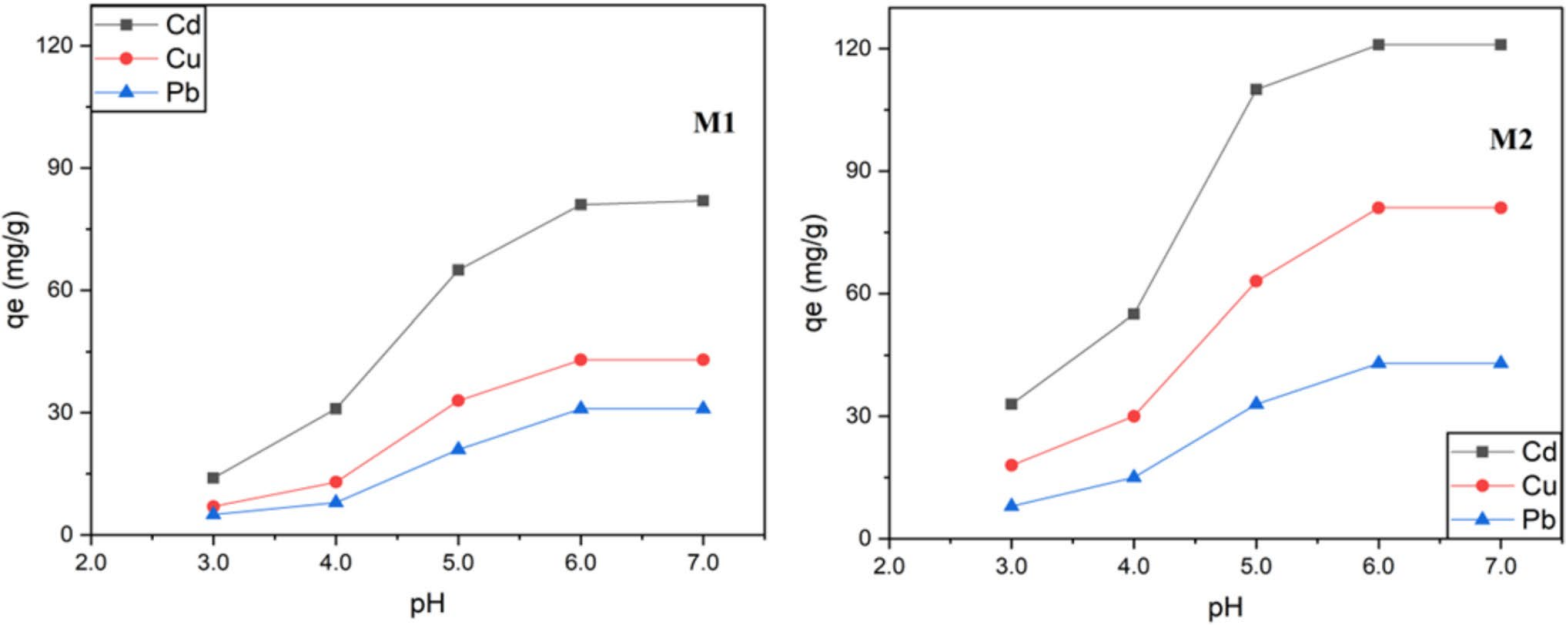
pH impact on the adsorption capacity of **M1** and **M2**. Adsorption conditions: 10 mg of **M1** or **M2** in 10 mL of an aqueous solution containing each metal ion at its optimal concentration (180 mg/L), at pH ranging from 3.0 to 7.0 for 30 min and at 298 K

So, the effect of pH in the adsorption efficiency of **M1** and **M2** was evaluated by performing the adsorption assays at aqueous solutions with pH ranging from 3.0 to 7.0 (Fig. 7). The experiments were performed with each metal ion at its optimal concentration value, for 30 min and at 298 K.

The results summarized in Fig. 7 show that both hybrid materials, **M1** and **M2**, exhibit similar performances against variations in the pH. The capacity of adsorption notably decreases below pH 4.0. This impact is commonly observed in the case of cadmium and lead ions (Abollino et al. 2003). The justification for this behavior is that the electrostatic attraction between the surface charge and the dissolved ions influences the surface complexation reactions. In fact, given that Cd(II) and Pb(II) have larger ionic radius (0.97 and 1.20 Å, respectively), they have lower charge density and are thus more influenced by the protonation of the surface groups, which results in a reduction of adsorption sites on the modified silica. The Cu(II) behavior, on the other hand, is most likely attributable to the structure of its aqueous form. In fact, [Cu(H_2_O)_6_]^2+^ possesses a tetragonal distortion due to the Jahn–Teller effect, which causes the octahedral structure to compress along the x- and y-axes (Nicholls 1974). Therefore, the binding of ligands along the x- and y-axis is supported, whereas the ligands along the z-axis are shielded from the Cu(II) ion by an additional electron. This contraction along the x- and y-axis results in a structure with four shorter bonds and two longer bonds, which partially hinders in part the binding of the Cu(II) with the silica surface groups. This effect is more pronounced when these groups are more protonated since the binding is only maintained in certain directions. As a consequence of all the above factors, the adsorption of Cu(II), Cd(II), and Pb(II) is tremendously diminished by cation exchange mechanism. Thus, the optimal pH to maximize the adsorption of both materials is pH 6.0.

#### Selectivity

Several factors can affect the selectivity of transition metal adsorption, such as the adsorbent structural and energetic attributes, as well as the physicochemical properties of the metal ions. These properties include ionic radius, atomic weight, electronegativity, complex geometry formation, and metal cation hydrolysis constants (Lu and Sorial 2009; des Ligneris et al. 2020).

**Fig. 8:**
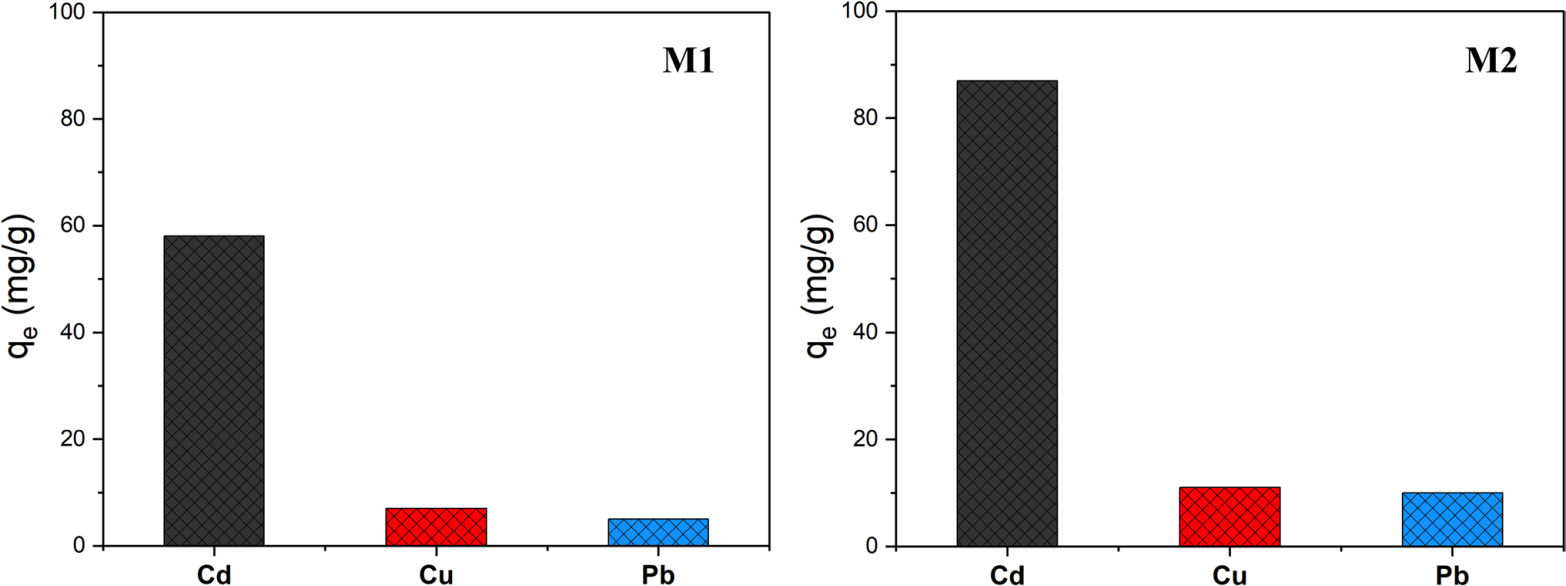
Effect of the presence of other metal ions on the adsorption efficiency of **M1** (left) and **M2** (right) to extract Cd(II): Adsorption conditions: 10 mg of **M1** and **M2** in 10 mL of an aqueous solution containing a mixture of the three metal ions (180 mg/L each) at pH 6.0 and 298 K for 30 min

In order to evaluate how the selectivity of **M1** and **M2** would be affected by the presence of other metal ions, a solution containing each adsorbent was maintained in contact with all metal ions, Cd(II), Cu(II), and Pb(II) (180 mg/L each) under the established optimal conditions (temperature at 298 K, a shaking time of 30 min, and pH set at 6.0). The outcomes of these experiments are depicted in Fig. 8.

**M1** and **M2** displayed notable selectivity against Cd(II) attaining an extraction of 58 and 87 mg/g, respectively. This value is due to the grafted ligand’s high coordination affinity towards it as reported by our group in the previous work (Draoui et al. 2023b). In fact, to immobilize the Cd(II) ion, two **L** branches are required. However, in the case of Cu(II) ion, it has been demonstrated that the coordination was maneuvered by three bidentate ligands, which makes it tougher to achieve. Nonetheless, the ability to retain Cd(II) experienced a decrease of *ca.* 28% for both adsorbents when compared to the adsorption capacity achieved through individual metal extraction. This reduction is primarily attributed to the adsorption of other metals, which leads to a decrease in available adsorption sites.

#### Reusability

**Fig. 9:**
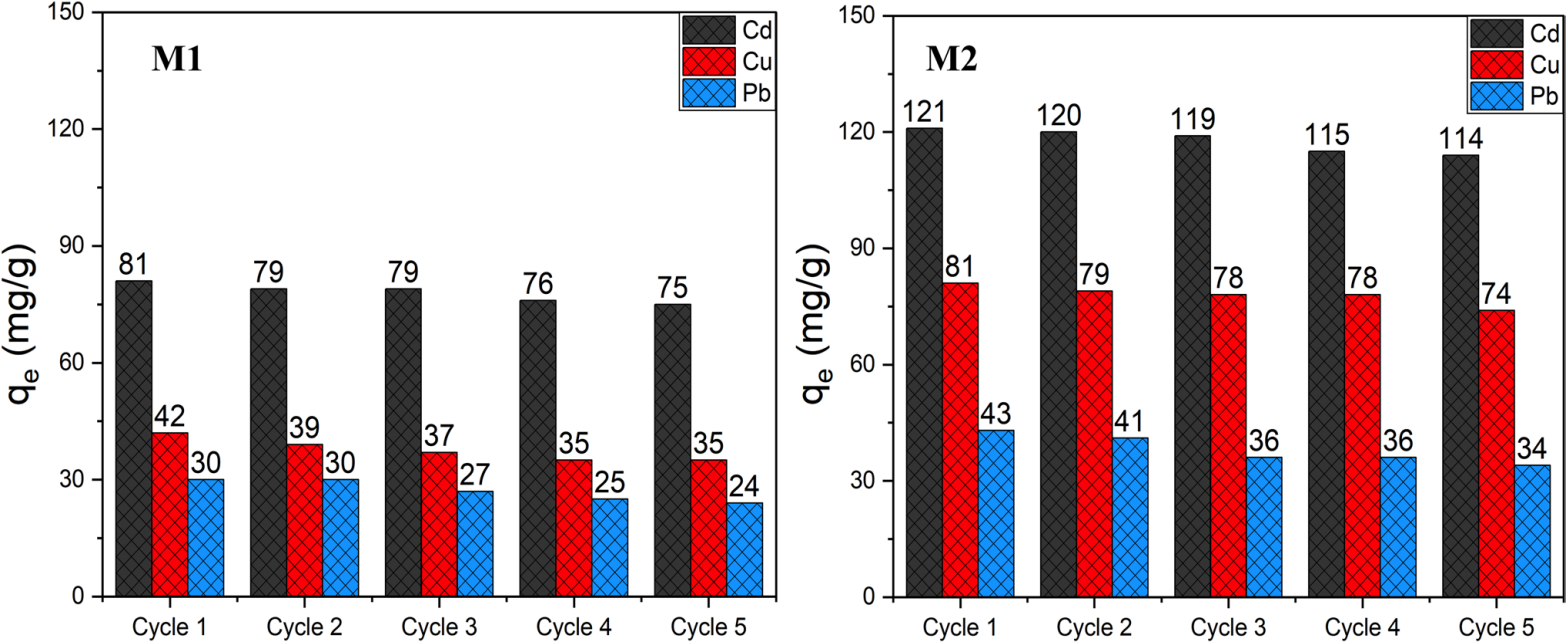
Regeneration performances of **M1** and **M2** over five cycles of metals extractions. Under the adsorption conditions of 10 mg of **M1** and **M2** in 10 mL of an aqueous solution containing each metal ion (180 mg/L) at pH 6.0 and 298 K for 30 min. After each cycle, the material was recovered after acidic treatment and reused

Numerous adsorbents can encompass most of the aforementioned advantages; however, their costly production and refinement can present a significant drawback. While adsorption capacity, contact time, and selectivity are substantial industrial objectives, the ability to regenerate and reuse adsorbents is critical to transforming them into cost-effective, scalable solutions. In line with this, the regeneration and reuse of each hybrid material were investigated. For this, after each use, the metal-laden adsorbents were maintained under agitation in an acidic aqueous solution (10 mL of nitric acid at 4%) for 2 h. Then, the resulting material was washed with water and subsequently dried for reuse. Figure 9 provides a summary of the number of cycles performed by **M1** and **M2** alongside their corresponding new extraction values.

Both hybrid materials demonstrated remarkable reliability and durability even after undergoing five usage cycles. Specifically, **M1** and **M2** exhibited an efficiency loss of less than 8% in their ability to extract their respective targeted Cd(II) ions. The modest reductions in the adsorption capacity of these adsorbents over multiple cycles are primarily attributed to the protonation of active surface sites by H^+^ ions in an acidic environment, leading to a reduction in available coordinating sites. In conclusion, these newly developed hybrid materials have demonstrated impressive recyclability, thereby substantiating their economic viability and competitive edge.

#### Application in real contaminated water

Throughout the assessment of various parameters, the valuable adsorbent characteristics of the newly synthesized hybrid materials **M1** and **M2** prompted us to explore their reliability with actual contaminated water samples collected from Marchica in Morocco’s Nador city, which is known for contamination with organic and inorganic pollutants (Aknaf et al. 2018).

Table 6 provides details on the heavy metal concentrations found in each sample, along with the quantities of heavy metals adsorbed by each material. Based on this collected data, it is evident that the utilized adsorbents proved to be highly reliable even at low metal concentrations. **M1** and **M2** demonstrated significant efficiencies of 57% and 64% of Cd(II) removal.

**Table 6:** Heavy metal removal from Marchica river water in 2022/09/19

Adsorbent	Metal	Concentration (mg/L)		% removed
		Found	Removed	
**M1**	Cd(II)	3.01	1.71	57%
	Cu(II)	15.17	3.32	22%
	Pb(II)	3.27	0.85	26%
**M2**	Cd(II)	3.01	2.02	67%
	Cu(II)	15.17	4.44	30%
	Pb(II)	3.27	0.89	27%

To facilitate a more comprehensive comparison with the selectivity experiments and to assess the influence of other potentially present ions in the samples, we introduced additional concentrations to the contaminated water, reaching 180 mg/L for each metal ion. The results of this study are summarized in Table 7, with the cutback column representing the percentage of efficiency lost compared to the values obtained in the selectivity test. Due to the potential interference of organic matter and alkaline ions in real water samples, the extracted concentrations were relatively lower compared to what was observed in the selectivity study. However, all materials performed admirably against Marchica water. This reduction in efficiency was barely noticeable for **M2** (7% performance cutback), as its selectivity proved to be exceptional.

**Table 7:** Heavy metal removal from Marchica river with additionally added metal concentrations (pH 6.0 at 298 K)

Adsorbent	Metal	Concentration (mg/L)		Cutback %
		Found	Removed	
**M1**	Cd(II)	180	47.21	19%
	Cu(II)	180	5.43	22%
	Pb(II)	180	2.81	44%
**M2**	Cd(II)	180	81.11	7%
	Cu(II)	180	10.19	7%
	Pb(II)	180	7.12	29%

#### Comparative study of adsorption capacity of Cd(II) to previous studies

Table 8 compares the adsorption capacities of the new hybrid materials **M1** and **M2** with those of the most effective reported adsorbents for Cd(II). **M1** and **M2**, with Cd(II) removal capacities of 81.40 and 121.26 mg/g, respectively, have significantly outperformed many previously reported studies. Their outstanding performance sets a new benchmark for Cd(II) removal.

**Table 8:** Comparative study of Cd(II) extraction capacity of the new hybrid materials **M1** and **M2** and those found with the literature

Adsorbent	Adsorption capacity (mg/g)	Reference
**M1**	81.40	This work
**M2**	121.26	This work
Silica-bipyridine	42.18	Radi et al. (2016)
Silica-tris(2-aminoethyl) amine	36.42	Huang et al. (2008)
SBA15-2-mercaptopyrimidine	111.28	Pérez-Quintanilla et al. (2006)
MSN	73	He et al. (2018)
Magadiite-Cyanex 272	48.45	Attar et al. (2018)
GL-cl-PAAm	54.95	Fosso-Kankeu et al. (2017)
Hybrid hydrogel composite	78.13	Pérez-Quintanilla et al. (2006)
Sol–gel hybrid sorbent	77.20	Wu and Yi (2013)
Sodium alginate	179.00	Wang et al. (2016)
LDH:H	80.00	Liñán-González et al. (2023)
SH-SiO2MS-Ca-Alg beads	70.68	Singh et al. (2022)

## Conclusion

In summary, this study was primarily focused on the synthesis of two novel environmentally friendly silica-based hybrid materials, with a definitive application as adsorbents for the removal of heavy metals ions, specifically Cd(II), Cu(II), and Pb(II). The characterization of **M1** and **M2** involved various methods that consistently confirmed the successful incorporation of the organic component **L** into both functionalized silica gel **Si-Cl** and **MCM-41-Cl**, respectively. Furthermore, the reliability and efficiency of these adsorbents were rigorously examined through the assessment of various adsorption parameters and fitting them to several models. In terms of the initial concentration parameter, **M1** demonstrated exceptional cadmium removal capabilities, while this efficiency significantly increased in the case of **M2**, owing to the larger specific surface area of **MCM-41**. Moreover, regarding the contact time parameter, both materials exhibited rapid metal removal, particularly noticeable in the case of **M1**, which mostly occurred within the first 5 min. Additionally, both materials displayed remarkable selectivity towards Cd(II) and sustained multiple usage cycles without any noteworthy degradation. In conclusion, it can be confidently stated that **M1** and **M2** are highly dependable for industrial-scale heavy metal extraction in aqueous solutions due to their ease of use, high cadmium extraction capacity, rapid metal elimination, and exceptional cost-effectiveness. The performance of these adsorbents against real contaminated samples from the Nador region further validates their practical utility.

## Supplementary Information

Below is the link to the electronic supplementary material.

Supplementary file1 (DOCX 3140 KB)

Supplementary file1 (PDF 542 KB)

## Data Availability

Data will be made available on request.
