# Supplementary material for: Tailoring selectivity and efficiency: pyrazolyl-1H-1,2,4-triazole MCM-41 and silica hybrid materials for efficient cadmium(II) removal from water
Source: Environ Sci Pollut Res Int. 2025 Apr 5;32(17):10984–1003. doi: 10.1007/s11356-025-36353-z (PMC12014845; doi:10.1007/s11356-025-36353-z)
Supplement: Supplementary file 1 — Supplementary file1 (DOCX 3140 KB) [file 11356_2025_36353_MOESM1_ESM.docx]

**Online Resource**

**Tailoring selectivity and efficiency: pyrazolyl-1*H*-1,2,4-triazole MCM-41 and silica hybrid materials for efficient Cadmium(II) removal from water**

Youssef Draoui ^a^, Smaail Radi ^a^, Amal El Mahdaoui ^a^, Mohamed El Massaoudi ^a^, Aurelian Rotaru ^b^, Yann Garcia ^c^, Maria do Amparo F. Faustino ^d^, Maria da Graça P. M. S. Neves ^d^, Nuno M. M. Moura ^d,*^

*^a^ LCAE, Department of Chemistry, Faculty of Science, University Mohamed I , P.O. Box 524, Oujda 60 000, Morocco.*

*^b^ Department of Electrical Engineering and Computer Science & Research Center MANSiD, “Stefan cel Mare” University, University Street, No. 13, Suceava 720229, Romania.*

*^c^ Institute of Condensed Matter and Nanosciences, Molecular Chemistry, Materials and Catalysis (IMCN/MOST), Université Catholique de Louvain, Place L. Pasteur 1, 1348 Louvain-la-Neuve, Belgium.*

*^d^ LAQV-REQUIMTE, Department of Chemistry, University of Aveiro, 3810-193 Aveiro, Portugal.*

* Corresponding authors: nmoura@ua.pt

**Content:**

**Equipments**…………………………………………………………………………………...S2

**Figure OR1.** Top: ATR-FTIR spectra of **M1** (A) and **M2** (B) and of the corresponding precursors. Bottom: the region between 1600 and 1200 cm^-1^ is zoomed (**A`** for **M1** and **B`** for **M2**) for detailed comparison………………………………………………….………S3
**Figure OR2.** Top: Solid state ^13^C NMR spectrum of precursors **Si-Cl** (left) and **MCM-41-Cl**(right).
Bottom: Solid state ^13^C NMR spectrum of **M1** (left) and **M2** (right)……………………….S4

**Figure OR3.** Thermogravimetric plots of **M1 (**left**)**, **M2 (**right) and of the corresponding precursors……………………………………………………………………………………...S5

**Equipments**

The characterization of silica particles was performed using scanning electron microscopy (SEM) with a Hitachi S4100 equipped with energy-dispersive spectroscopy. The specific surface area and pore size distribution were determined using the Brunauer-Emmett-Teller (BET) and Barrett-Joyner-Halenda (BJH) methods on a Micromeritics Gemini 2380 surface area analyzer, with a sample weight of approximately 50 mg. Solid-state ^13^C Nuclear Magnetic Resonance (NMR) spectra were recorded on a Bruker Avance III 400 spectrometer. Elemental analysis was conducted using a LECO CHNS-932 apparatus. Attenuated Total Reflectance Fourier Transform Infrared (ATR-FTIR) spectra were obtained using a FT Mattson 7000 Galaxy series spectrophotometer. Nitrogen adsorption-desorption measurements were carried out with a Thermoquest Sorpsomatic 1990 analyzer. Mass loss determinations were performed in a 90:10 oxygen/nitrogen atmosphere using a TGA Q50 V6.7 Build 203 instrument at a heating rate of 10 °C/min. Atomic absorption measurements were conducted using a Spectra Varian A.A. 400 spectrophotometer. Calibration curves were prepared using commercially pure 1000 ppm metal standard solutions in 2% nitric acid.


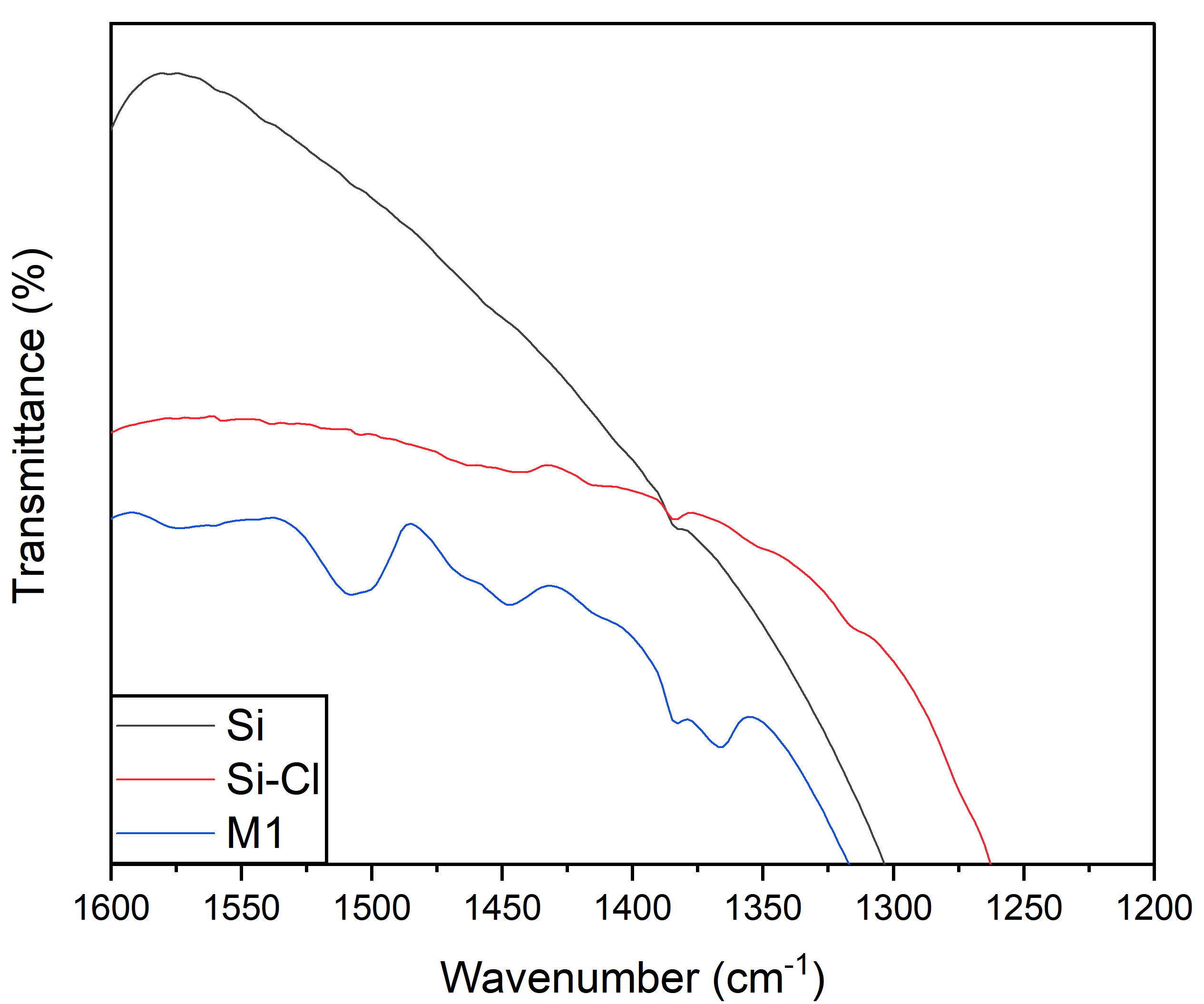

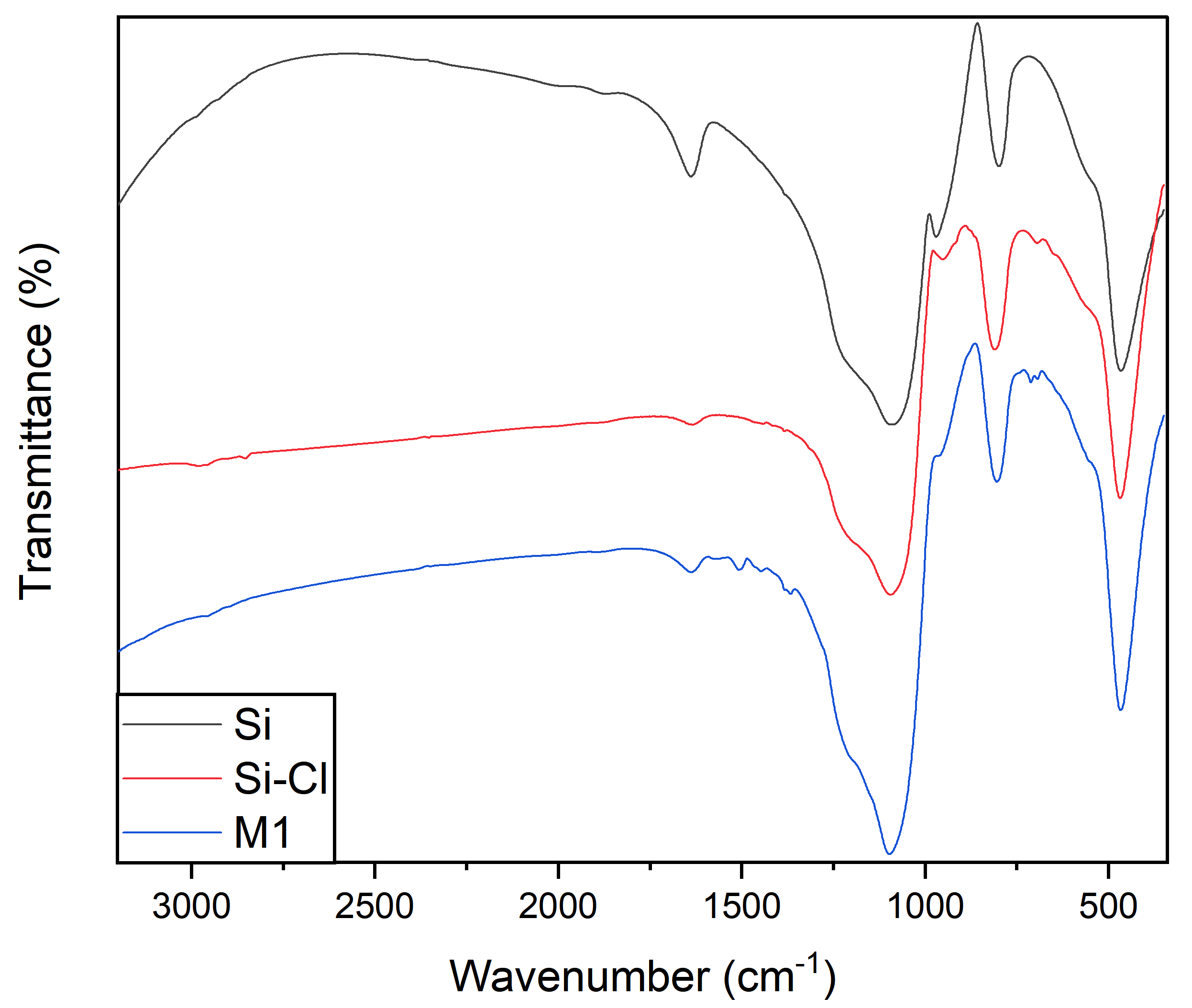

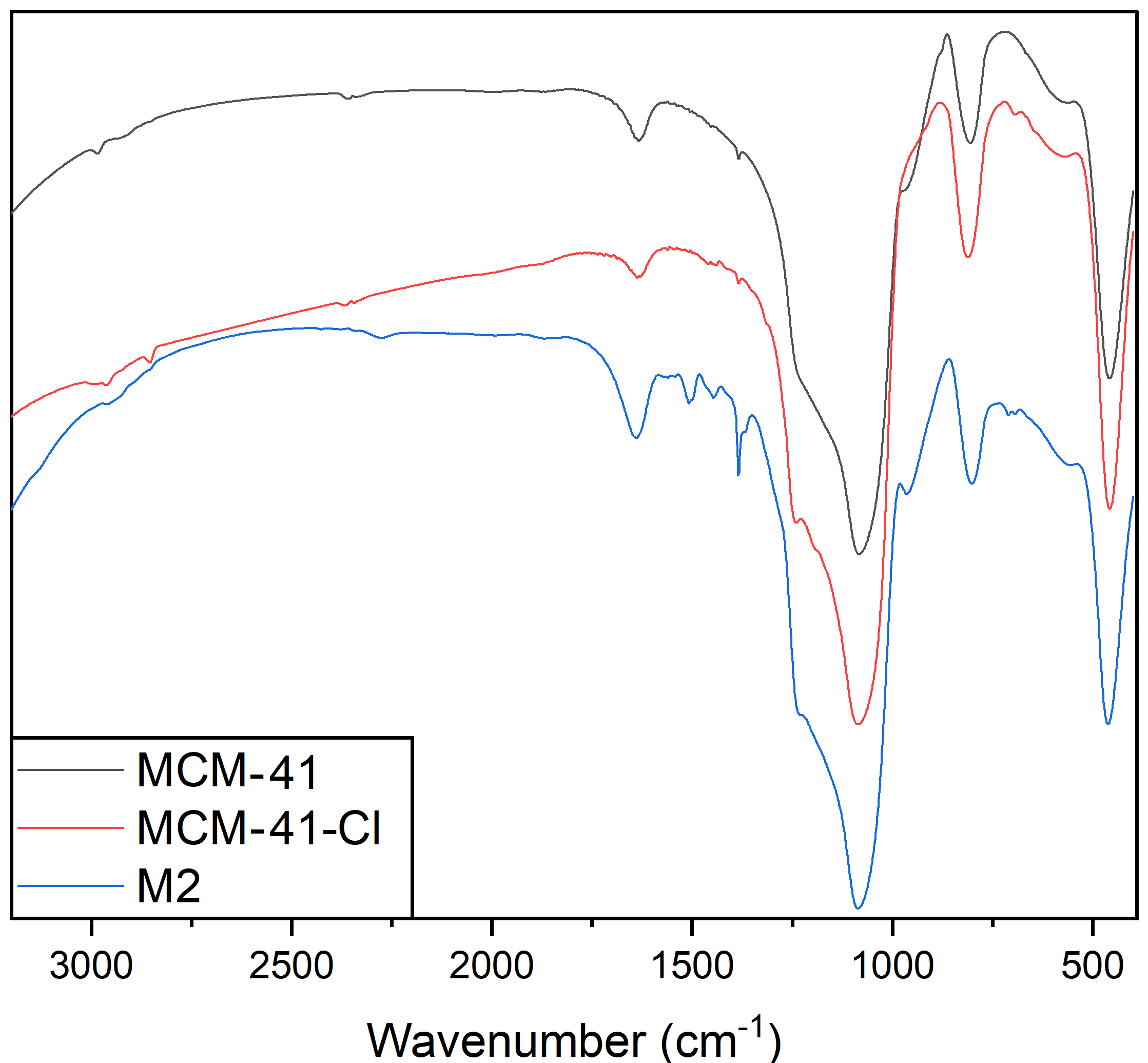

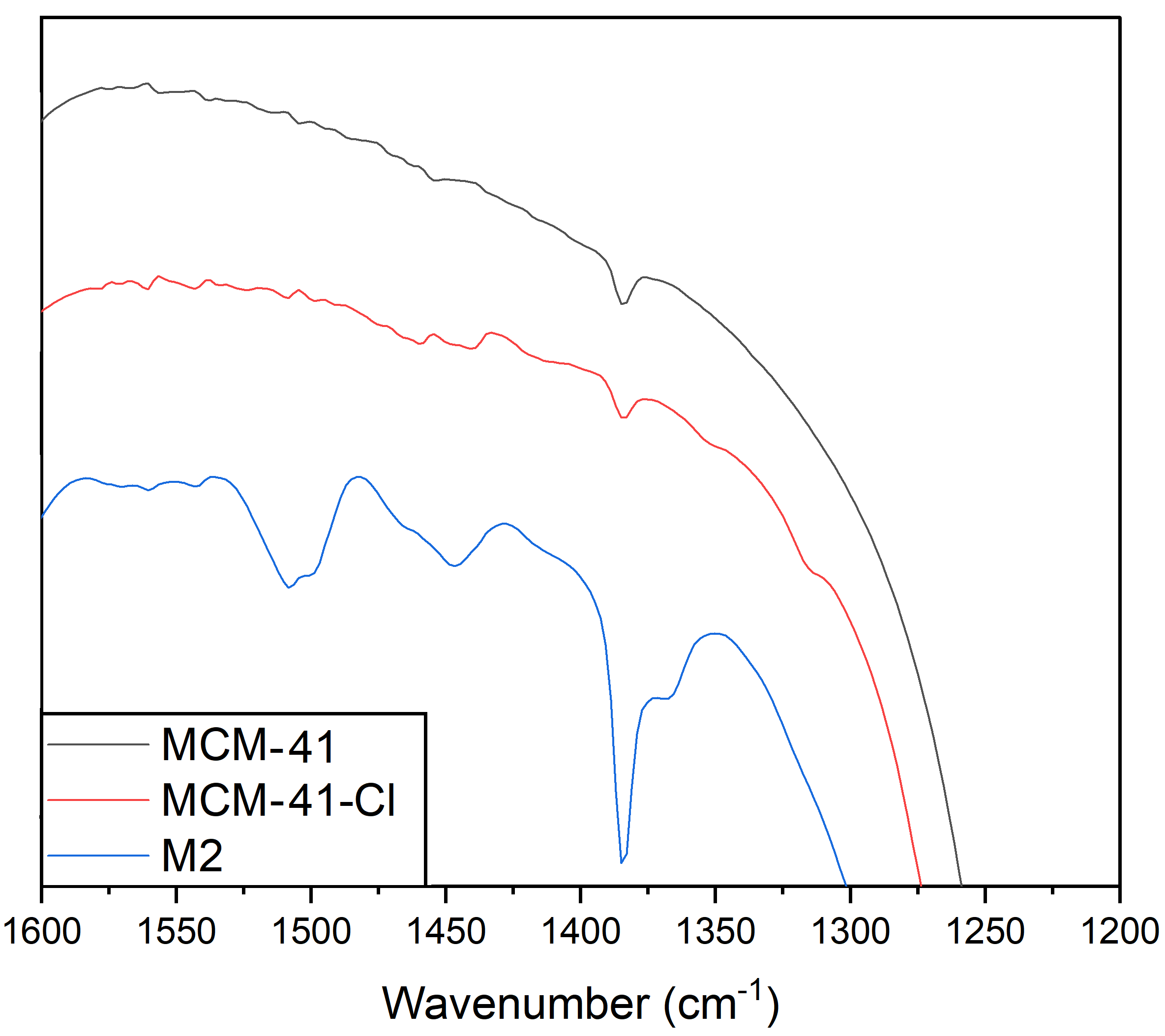


Zoomed

Zoomed

**(B´)**

**(A´)**

**(A)**

**(B)**

**Figure OR1.** Top: ATR-FTIR spectra of **M1** (A) and **M2** (B) and of the corresponding precursors. Bottom: the region between 1600 and 1200 cm^-1^ is zoomed (**A`** for **M1** and **B`** for **M2**) for detailed comparison.

**Si-Cl**

**MCM-41-Cl**

**M1**

**M2**

**Figure OR2.** Top: Solid state ^13^C NMR spectrum of precursors **Si-Cl** (left) and **MCM-41-Cl** (right). Bottom: Solid state ^13^C NMR spectrum of **M1** (left) and **M2** (right).


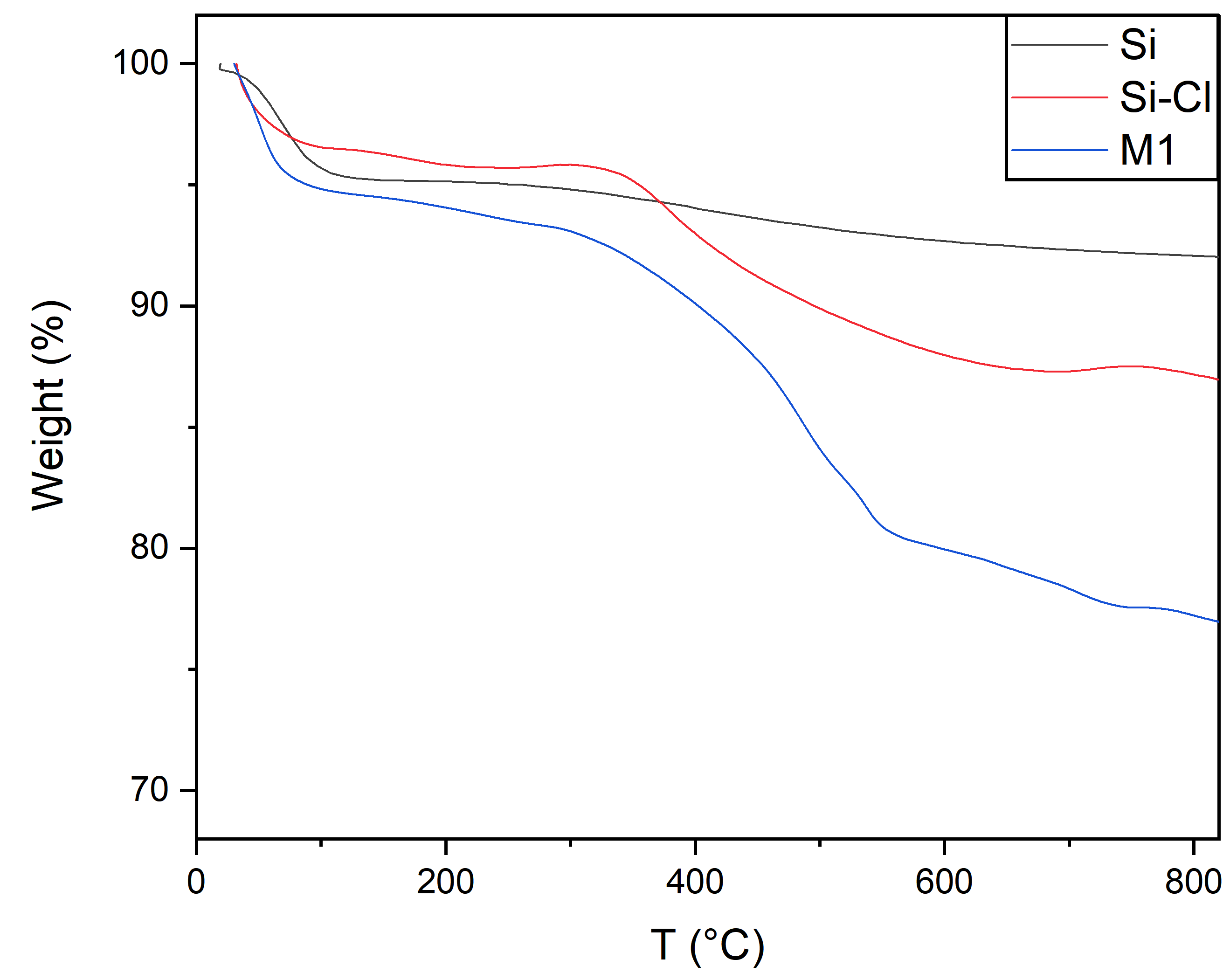

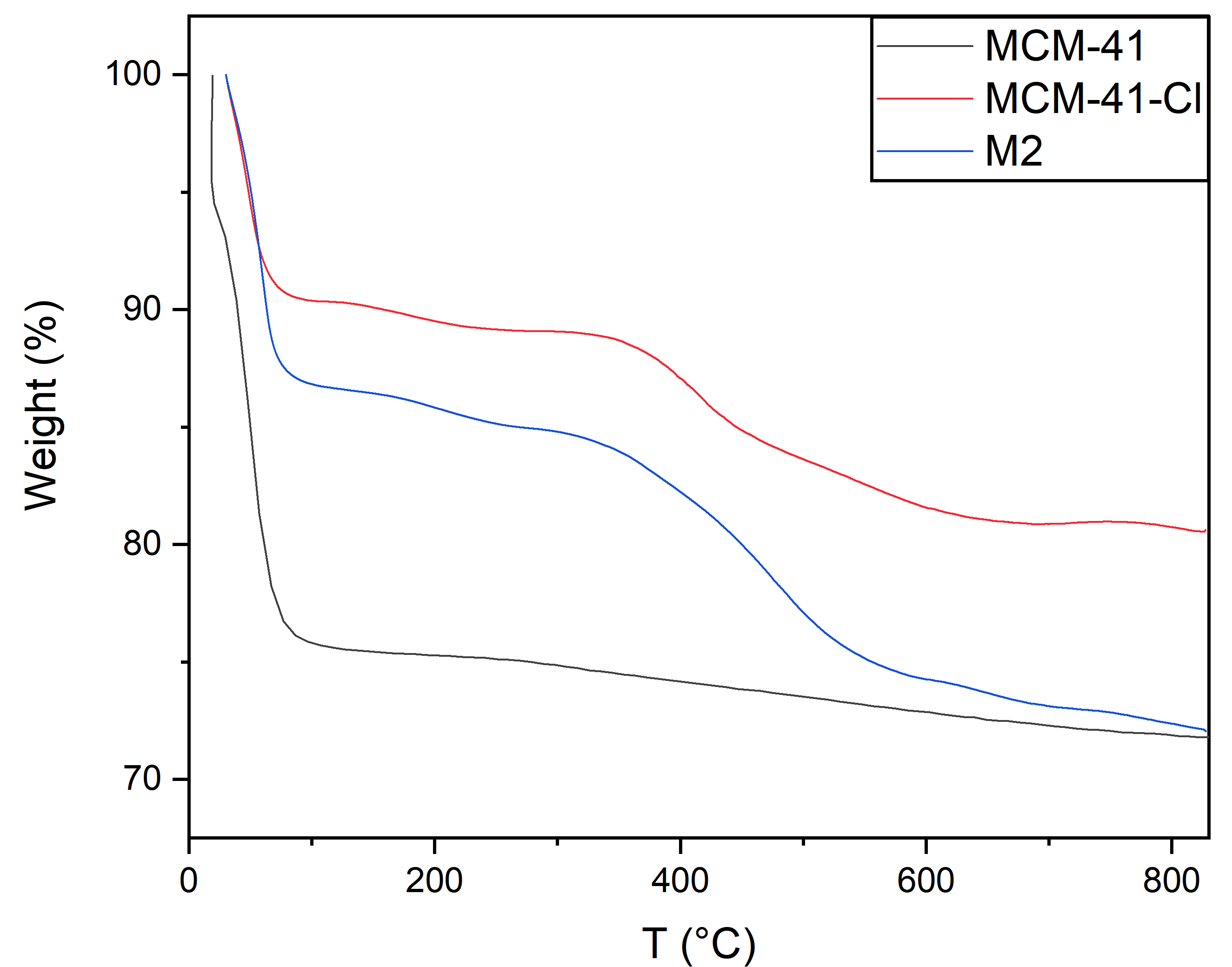


**Figure OR3**. Thermogravimetric plots of **M1 (**left**)**, **M2 (**right) and of the corresponding precursors.
