# Supplementary material for: Tailoring selectivity and efficiency: pyrazolyl-1H-1,2,4-triazole MCM-41 and silica hybrid materials for efficient cadmium(II) removal from water
Source: Environ Sci Pollut Res Int. 2025 Apr 5;32(17):10984–1003. doi: 10.1007/s11356-025-36353-z (PMC12014845; doi:10.1007/s11356-025-36353-z)
Supplement: Supplementary file 2 — Supplementary file1 (PDF 542 KB) [file 11356_2025_36353_MOESM2_ESM.pdf]

Online Resource

Tailoring selectivity and efficiency: pyrazolyl-1*H*-1,2,4-triazole MCM-41 and silica hybrid materials for efficient Cadmium(II) removal from water

Youssef Draoui [a], Smaail Radi [a], Amal El Mahdaoui [a], Mohamed El Massaoudi [a], Aurelian Rotaru [b], Yann Garcia [c], Maria do Amparo F. Faustino [d], Maria da Graça P. M. S. Neves [d], Nuno M. M. Moura [d,*]

[a] *LCAE, Department of Chemistry, Faculty of Science, University Mohamed I , P.O. Box 524, Oujda 60 000, Morocco.*

[b] *Department of Electrical Engineering and Computer Science & Research Center MANSiD, "Stefan cel Mare" University, University Street, No. 13, Suceava 720229, Romania.*

[c] *Institute of Condensed Matter and Nanosciences, Molecular Chemistry, Materials and Catalysis (IMCN/MOST), Université Catholique de Louvain, Place L. Pasteur 1, 1348 Louvain-la-Neuve, Belgium.*

[d] *LAQV-REQUIMTE, Department of Chemistry, University of Aveiro, 3810-193 Aveiro, Portugal.*

* Corresponding authors: nmoura@ua.pt

Content:

Equipments……………………………………………………………………………………...S2

Figure OR1. X-ray diffraction patterns of **M1** (A) and **M2** (B) in comparison to their precursors…………………………………………………………………………………..…S3

Figure OR2. Top: Solid state ^{13}C NMR spectrum of precursors **Si-Cl** (left) and **MCM-41-Cl**(right). Bottom: Solid state ^{13}C NMR spectrum of **M1** (left) and **M2** (right)…………………………………………………………………………………..….S4

Figure OR3. Thermogravimetric plots of **M1** (left), **M2** (right) and of the corresponding precursors……………………………………………………………………………………...S5

Equipments

The characterization of silica particles was performed using scanning electron microscopy (SEM) with a Hitachi S4100 equipped with energy-dispersive spectroscopy. The specific surface area and pore size distribution were determined using the Brunauer-Emmett-Teller (BET) and Barrett-Joyner-Halenda (BJH) methods on a Micromeritics Gemini 2380 surface area analyzer, with a sample weight of approximately 50 mg. Solid-state ^{13}C Nuclear Magnetic Resonance (NMR) spectra were recorded on a Bruker Avance III 400 spectrometer. Elemental analysis was conducted using a LECO CHNS-932 apparatus. Attenuated Total Reflectance Fourier Transform Infrared (ATR-FTIR) spectra were obtained using a FT Mattson 7000 Galaxy series spectrophotometer. Powder X-ray diffraction (PXRD) patterns were collected on a D8-Advance diffractometer (Bruker, Germany) with Cu Kα radiation (λ = 1.5148Å) operating at 40 kV and 30 mA. Nitrogen adsorption-desorption measurements were carried out with a Thermoquest Sorpsomatic 1990 analyzer. Mass loss determinations were performed in a 90:10 oxygen/nitrogen atmosphere using a TGA Q50 V6.7 Build 203 instrument at a heating rate of 10 °C/min. Atomic absorption measurements were conducted using a Spectra Varian A.A. 400 spectrophotometer. Calibration curves were prepared using commercially pure 1000 ppm metal standard solutions in 2% nitric acid.

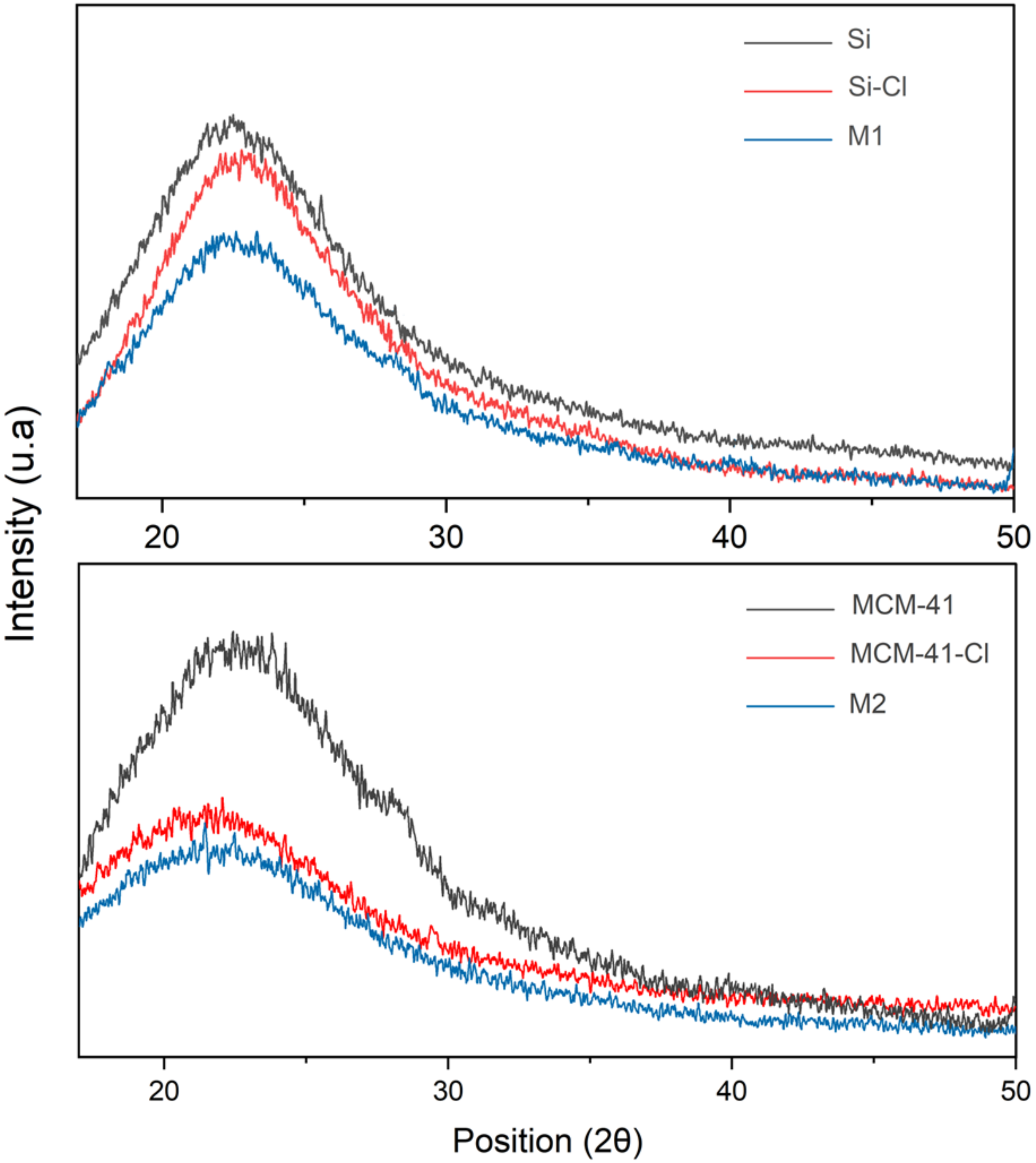

Figure OR1. X-ray diffraction patterns of **M1**(A) and **M2** (B) in comparison to their precursors.

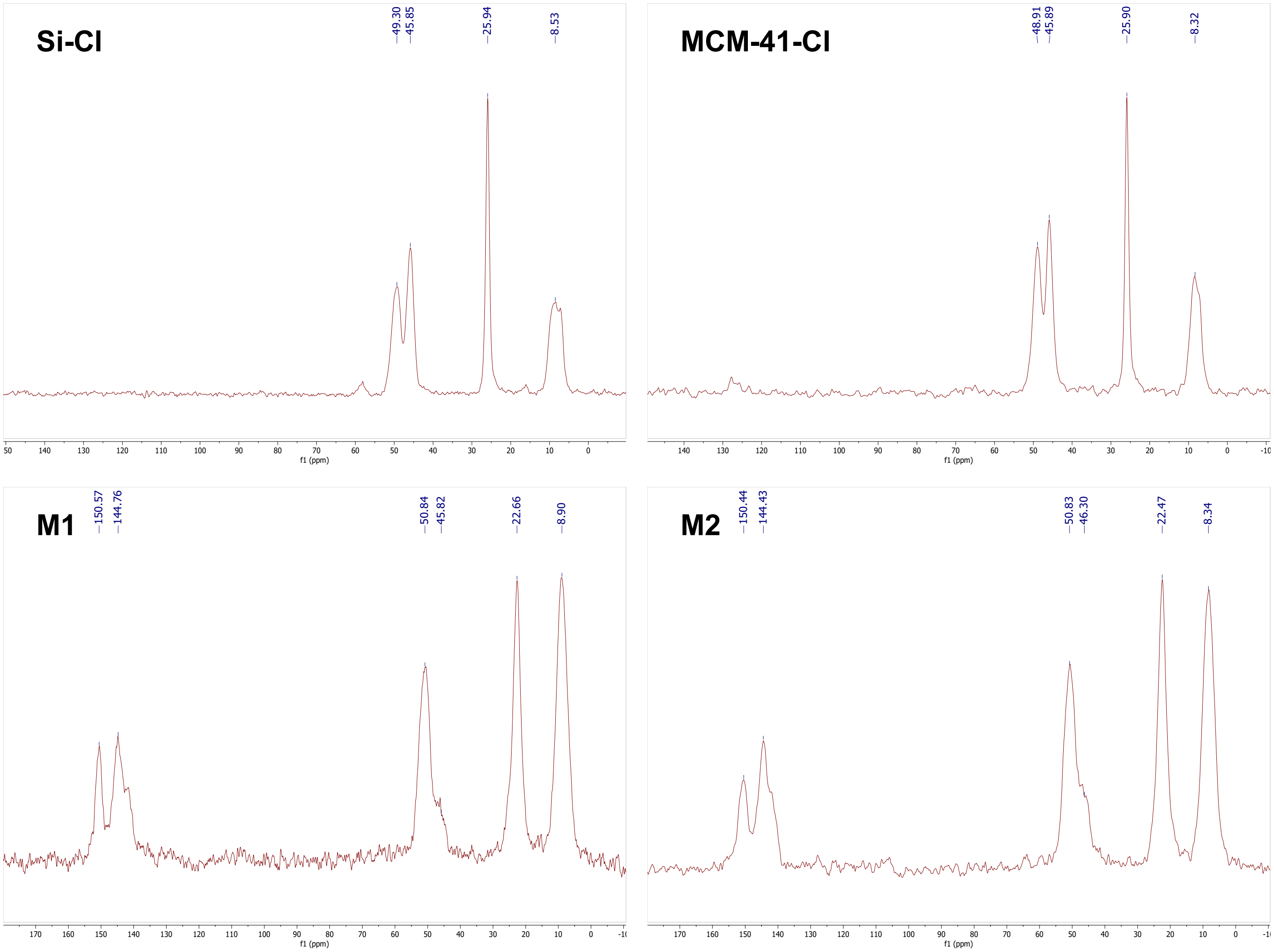

Figure OR2. Top: Solid state ^{13}C NMR spectrum of precursors **Si-Cl** (left) and **MCM-41-Cl** (right). Bottom: Solid state ^{13}C NMR spectrum of **M1** (left) and **M2** (right).

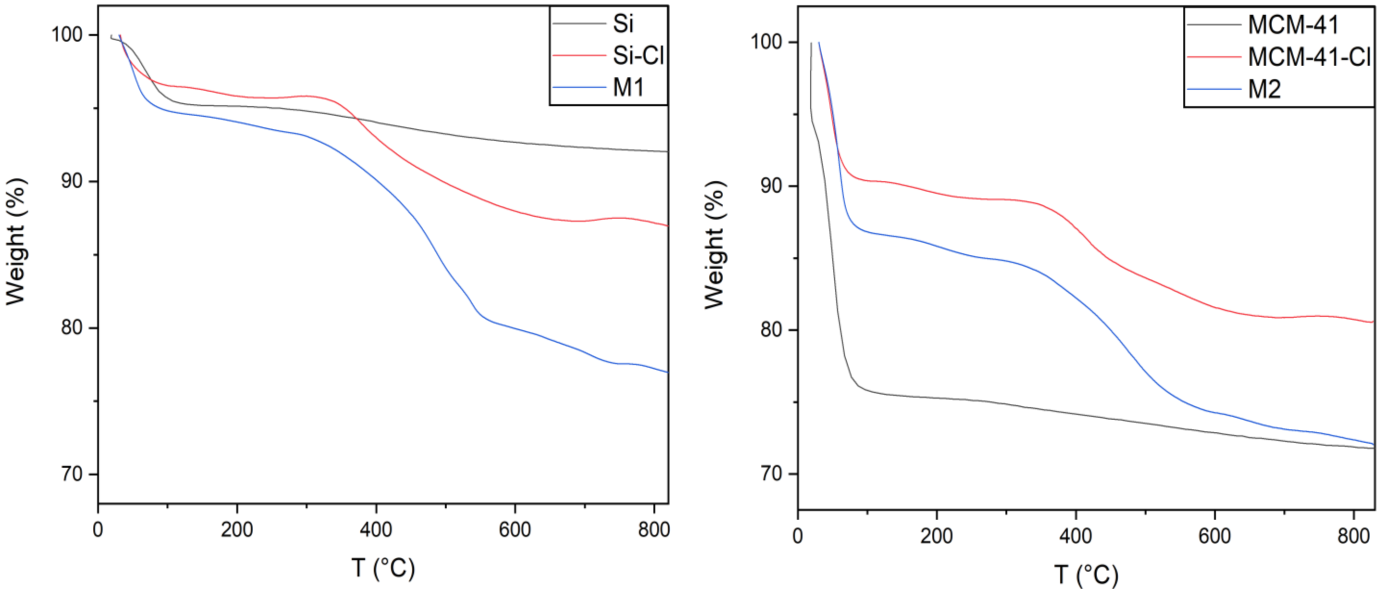

Figure OR3. Thermogravimetric plots of **M1 (**left**)**, **M2 (**right) and of the corresponding precursors.
